# Ultrasound‐Mediated Biotransfection of Engineered Bone Marrow Mesenchymal Stem Cells in Treated Bone Defects through Intracellular Cavitation

**DOI:** 10.1002/advs.202503196

**Published:** 2025-07-30

**Authors:** Zhili Xu, Huijuan Xin, Yu Wang, Renhao Xu, Yanni He, Meijun Zhou, Zhengqiang Yuan, Hongmei Liu

**Affiliations:** ^1^ Department of Ultrasound Institute of Ultrasound in Musculoskeletal Sports Medicine The Affiliated Guangdong Second Provincial General Hospital of Jinan University Guangzhou 510317 China; ^2^ School of Biomedical and Pharmaceutical Sciences Guangdong University of Technology Guangzhou 510006 China

**Keywords:** bone defects, bone mesenchymal stem cells, gas vesicles, gene transfection, ultrasound‐targeted microbubble destruction

## Abstract

The lack of stem cells and difficulty in osteogenic differentiation are the primary challenges to treating bone defects. Stem cell gene therapy can efficiently replenish the number of stem cells and facilitate bone differentiation, but its security and efficacy remain challenging. The traditional ultrasound‐targeted microbubble destruction (UTMD) technology with extracellular cavitation for gene transfection is safe but inefficient. Consequently, gas vesicles extracted from *Halobacterium NRC‐1* are used as carriers, incorporating nuclear localization signal, polyethyleneimine, and plasmid bone morphogenetic protein 2 (pBMP2). Then followed by internalization into bone marrow mesenchymal stem cells (BMSCs) to produce engineered BMSCs, which exhibit significant capacity of lysosome escape and nuclear targeting. The permeability of the nuclear membrane is substantially enhanced by low‐intensity pulsed ultrasound through intracellular cavitation, thereby increasing plasmid nuclear translocation efficiency and gene transfection efficiency by 284.7% and 131.6%, respectively, compared to conventional UTMD techniques. Besides, the expression of BMP2 is maintained for 21 days, promoting osteogenic differentiation of BMSCs and enhancing bone defect repair. In conclusion, this study provides a more secure, efficient, and regulated approach to BMSCs gene therapy for bone defects.

## Graphical abstract

Ultrasound‐mediated biotransfection through intracellular cavitation increases plasmid nuclear translocation and gene transfection efficiency by 284.7% and 131.6% in engineered bone marrow mesenchymal stem cells (BMSCs) compared to traditional ultrasound‐targeted microbubble destruction techniques. The maintained bone morphogenetic protein 2 expression for 21 days promotes osteogenic differentiation of BMSCs and improves the repair of bone defects.

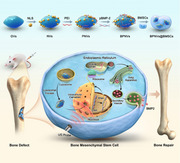

## 1 Introduction

Bone defects involve loss of bone structure due to accidents, injuries, bone tumors, tuberculosis, and other factors.^[^ ^1^ ^]^ While minor defects heal naturally, severe cases involving significant loss of bone, infection, metabolic disorders, or aging often result in incomplete regeneration or nonunion fractures.^[^ ^2^ ^]^ Bone graft is the general treatment. Globally, 4 million people require bone transplantation or replacement surgery each year.^[^ ^3^ ^]^ However, it is invasive, slow‐healing, and struggles with stem cell shortages and osteogenic differentiation challenges.^[^ ^4^ ^]^ Therefore, enhancing stem cell supplementation and promoting osteogenic differentiation are significant challenges in bone defect therapy.

Stem cell gene therapy has promising clinical applications, utilizing gene transfection to modify stem cells and reintroduce them into the patient.^[^ ^5^ ^]^ Compared to traditional bone graft, it not only reduces trauma but also enhances stem cell quantity and osteogenic differentiation.^[^ ^6^ ^]^ Despite advances in basic research, clinical application remains challenging, particularly due to safety concerns.^[^ ^7^ ^]^ Current transfection strategies include biological (viral transfection, phage‐mediated transduction, etc.), chemical (lipofection, calcium phosphate‐DNA coprecipitation, diethylaminoethyl‐dextran‐mediated transfection, etc.), and physical methods (electroporation, particle bombardment, microinjection, etc.).^[^ ^8^ ^]^ Viruses are the traditional gene carriers in biological transfection approaches with high transfection efficiency.^[^ ^9^ ^]^ Falkenhagen et al. transfected HEK‐293T cells with lentivirus, achieving a transfection rate close to 100%.^[^ ^10^ ^]^ Even so, clinical use of viruses as carriers raises ethical and safety concerns.^[^ ^11^ ^]^ Traditional physical or chemical transfection techniques often induce cytotoxicity, cellular damage, and instability.^[^ ^12^ ^]^ For example, high‐voltage electroporation and laser irradiation may cause cell necrosis, apoptosis, and permanent cell damage. Chemical transfection agents may be cytotoxic and cause cell inflammation.^[^ ^13^ ^]^ Thus, developing a biocompatible gene transfection technology is critical for overcoming these challenges and advancing the clinical application of stem cell gene therapy.

Ultrasound‐targeted microbubble destruction (UTMD) is a noninvasive gene transfection technique with promising applications.^[^ ^14^ ^]^ It operates by using ultrasound to induce cavitation in gas‐containing cavitation nuclei like microbubbles and nanobubbles, enhancing cell membrane permeability to facilitate gene delivery.^[^ ^15^ ^]^ Despite its advantages in safety and controllability, UTMD's transfection efficiency remains low (10%), primarily due to limited nuclear membrane permeability.^[^ ^16^ ^]^ Recent advancements, such as introducing cavitation nuclei intracellularly, have improved HEK‐293T cell transfection efficiency to 46.7% compared to 11.7% with extracellular cavitation.^[^ ^17^ ^]^ However, transfection efficiency varies significantly across cell types, with HEK‐293T cells showing high sensitivity (80%), while mesenchymal stem cells (e.g., Wharton's jelly mesenchymal stem cells) exhibit low sensitivity (20%).^[^ ^18^ ^]^ Thus, improving the transfection efficiency of insensitive cells like mesenchymal stem cells remains a major challenge.

The intracellular transport of gene‐loaded nanoparticles involves a complex process, with only one‐fourteenth of them successfully reaching the nucleus due to lysosomal degradation and cytoplasmic barriers.^[^ ^19^ ^]^ Consequently, how to increase the nuclear translocation efficiency of exogenous genes is a major challenge in gene therapy. Polyetherimide (PEI) is a cationic polymer with a high density of nitrogen atoms, which can escape from the lysosome through the proton‐sponge effect.^[^ ^20^ ^]^ It binds to lysosome‐associated membrane protein‐1, facilitating lysosomal escape via osmotic swelling and membrane rupture.^[^ ^21^ ^]^ Similarly, nuclear localization signal (NLS) is a short peptide with positively charged residues.^[^ ^22^ ^]^ It can promote gene delivery to the nucleus by protecting DNA from lysosomal nucleases by compressing DNA.^[^ ^23^ ^]^ Moreover, NLS can be recognized by cytoplasmic transport receptors and facilitate gene translocation through nuclear import activity.^[^ ^24^ ^]^ Decrease the distance between cavitation nuclei and membranes, thereby increasing the chance of cavitation. Therefore, combining intracellular cavitation with PEI and NLS will offer a novel and promising strategy for improving gene therapy efficiency in stem cells.

**Figure 1:**
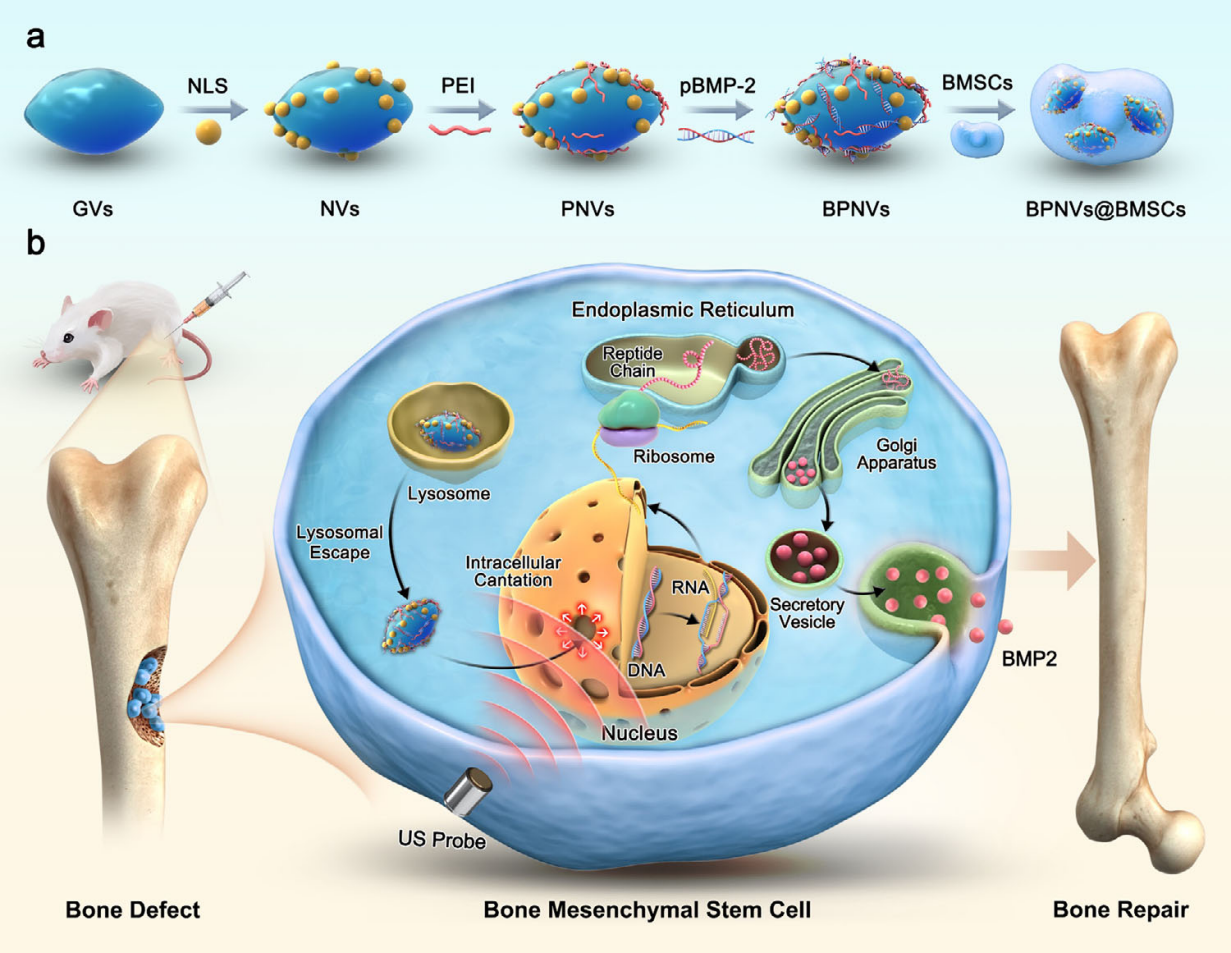
Schematic design of ultrasound‐mediated biotransfection of engineered BMSCs. a) NLS, PEI, and pBMP2 were loaded into the GVs to create BPNVs. These were subsequently incubated with BMSCs to form BPNVs@BMSCs. b) BPNVs were transported by NLS around the nuclear membrane after escape from lysosomes. BPNVs@BMSCs were treated with LIPUS stimulation to induce intracellular cavitation, which enlarged nuclear pores and enhanced both transcription and translation of pBMP2. The ensuing BMP2 facilitated the osteogenic differentiation of BMSCs and the repair of bone defects.

We developed engineered bone marrow mesenchymal stem cells (BPNVs@BMSCs) to enhance stem cell gene therapy for bone defects. By coating gas vesicles (GVs) extracted from *Halobacterium NRC‐1* with PEI and NLS, we combined with bone morphogenetic protein 2 plasmid (pBMP2) to create pBMP2/PEI/NLS‐loaded GVs (BPNVs).^[^ ^25^ ^]^ Then, BPNVs were internalized by bone marrow mesenchymal stem cells (BMSCs), guided to the nuclear membrane via NLS after lysosomal escape. Low‐intensity pulsed ultrasound (LIPUS) induced intracellular cavitation, enhancing nuclear membrane permeability and facilitating pBMP2 entry for transcription and translation. NLS optimized sonoporation efficiency by reducing the distance between GVs and the nuclear membrane, allowing for effective sound pore generation. When grafted into rat bone defects, BPNVs@BMSCs produced BMP2, promoting osteogenic differentiation of BMSCs and accelerating bone repair. This ultrasound‐mediated biotransfection strategy enhances BMSCs supplementation and promotes their directional osteogenic differentiation, offering a safer and more effective approach for stem cell gene therapy in bone defects (**Figure** **1**).

## 2 Results and Discussion

### 2.1 Fabrication and Characterization of Engineered BMSCs

**Figure 2:**
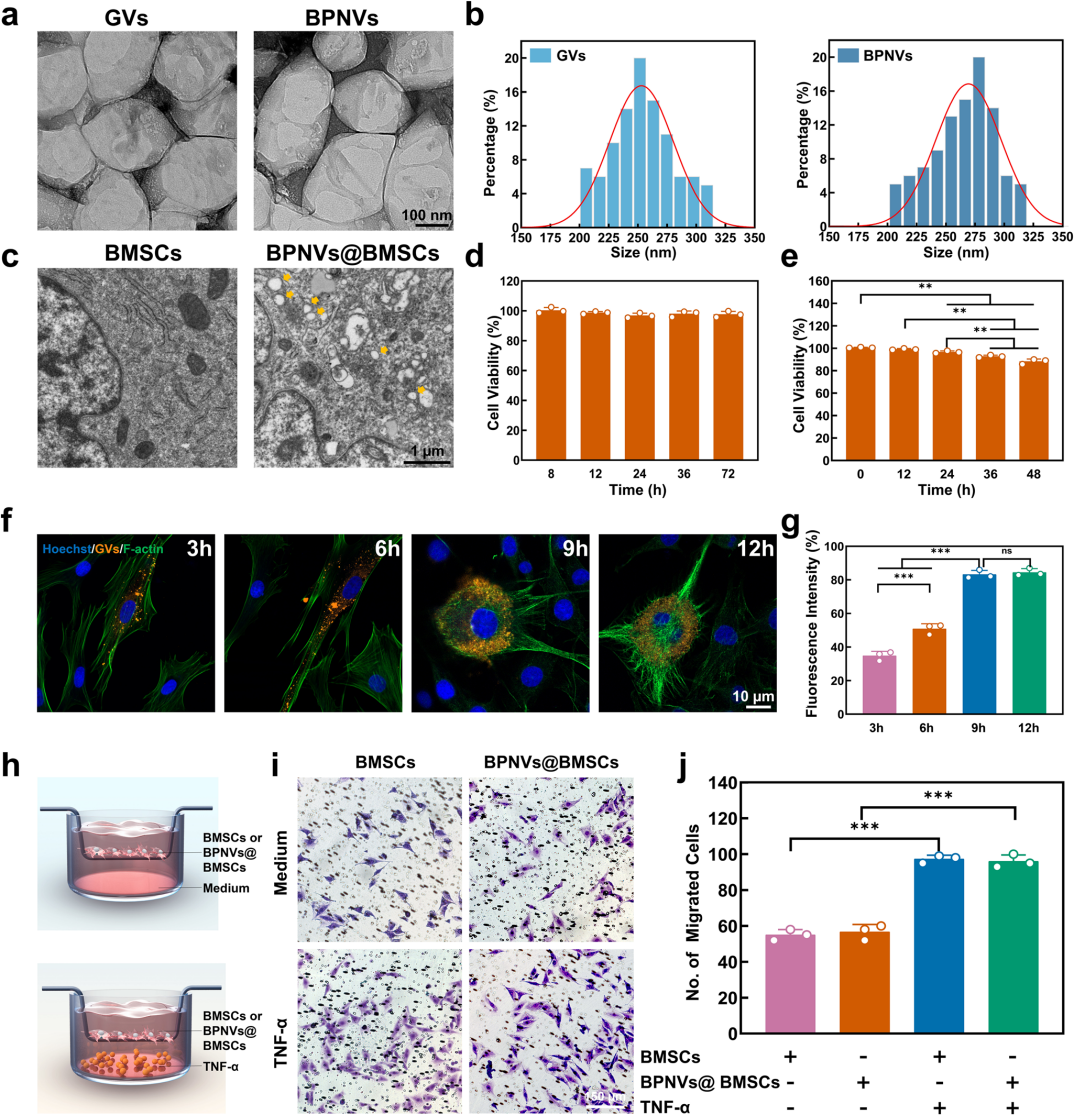
Characterization of GVs, BPNVs, and BPNVs@BMSCs. a) TEM images of GVs and BPNVs. b) Size distribution analysis of GVs and BPNVs (*n*  =  100). c) TEM images of BMSCs and BPNVs@BMSCs. Yellow arrows denote BPNVs. d) Percentages of viable BMSCs co‐incubated with GVs (OD_500_ = 1, *n*  =  3). e) Percentages of viable BMSCs co‐incubated with BPNVs (*n*  =  3). f,g) Confocal microscopy images (f) and quantitative analysis (g) of BPNVs@BMSCs after coincubation with BPNVs (*n*  =  3). h) Schematic illustration of transwell chambers for assaying in vitro inflammatory chemotactic capability. BMSCs or BPNVs@BMSCs were seeded into the upper chambers. The lower chambers were added with TNF‐α or sterile culture medium. The migrated cells on the lower side of the transwell membranes were stained with crystal violet (purple), followed by being counted using the light microscope. i,j) Representative microscopy images (i) and quantitative analysis (j) of migrating BMSCs and BPNVs@BMSCs after coincubation with or without TNF‐α for 12 h (*n*  =  3). ^*^ *p* < 0.5, ^**^ *p* < 0.01, ^***^ *p* < 0.001 by one‐way analysis of variance (ANOVA) (d,e,g) with Bonferroni correction and analysis of variance of factorial design (j); ns = no significance.

GVs were extracted from *Halobacterium NRC‐1* and coated with NLS and PEI to obtain PEI/NLS‐loaded GVs (PNVs) (Figure 1a; Figure S1, Supporting Information).^[^ ^25b^ ^]^ The potential of NLS‐loaded GVs (NVs) increased in proportion to the dosage of NLS (Figure S2a, Supporting Information). However, its negatively charged surface makes it inappropriate as a gene carrier.^[^ ^26^ ^]^ The incorporation of PEI enables the transformation of negative potential into positive potential, facilitating gene loading (Figure S2b,c, Supporting Information).^[^ ^27^ ^]^ Subsequently, pBMP2 was added to generate BPNVs (Figure S3, Supporting Information). Quantitative analysis shows that PNVs with 20 µg PEI can carry 10 µg pBMP2 (Figure S4, Supporting Information). As Figure S5 (Supporting Information) shown, the typical spectral peaks at 581 nm (GVs), 656 nm (PEI), and 654 nm (NLS) were clearly visible in the spectra of the BPNVs. Furthermore, their spectral images overlapped spatially, confirming the successful modification of GVs, PEI, and NLS on the surfaces of the GVs. The transmission electron microscope (TEM) images revealed that both GVs and BPNVs were spindle‐shaped with homogeneous particle sizes of ≈254.5 ± 27.1 and 265.3 ± 28.3 nm, respectively (**Figure** **2**a,b).

BPNVs were easily internalized by BMSCs, with the ingested contents primarily located within the cytoplasm (Figure 2c). GVs, NLS, PEI, and BPNVs exhibited complete biosafety, with the cell activity of BMSCs remaining above 90% following co‐incubation for 48 h. (Figure 2d,e; Figure S6, Supporting Information). Furthermore, the BMSCs phagocytosed the BPNVs in a time‐dependent manner (Figure 2f,g). Immunohistochemical staining demonstrated that GVs and PNVs had no substantial influence on the osteogenesis, adipogenesis, and chondrogenesis of BMSCs (Figure S7, Supporting Information). To determine whether BPNVs@BMSCs maintain a chemotactic movement toward inflammation, we cultured BMSCs or BPNVs@BMSCs in transwell inserts in the presence or absence of tumor necrosis factor‐α (TNF‐α) (Figure 2h).^[^ ^28^ ^]^ While very few control or gene‐loaded cells migrated through the transwell membrane when the receiver wells only contained sterile culture medium, the number of migrating cells increased significantly when TNF‐α was present in the receiver wells (Figure 2i). No significant difference was observed in the migratory capacities between the BMSCs and BPNVs@BMSCs (Figure 2j), indicating that the BPNVs did not affect the inherent inflammatory chemotactic properties of the BMSCs.

### 2.2 Lysosomal Escape of BPNVs

**Figure 3:**
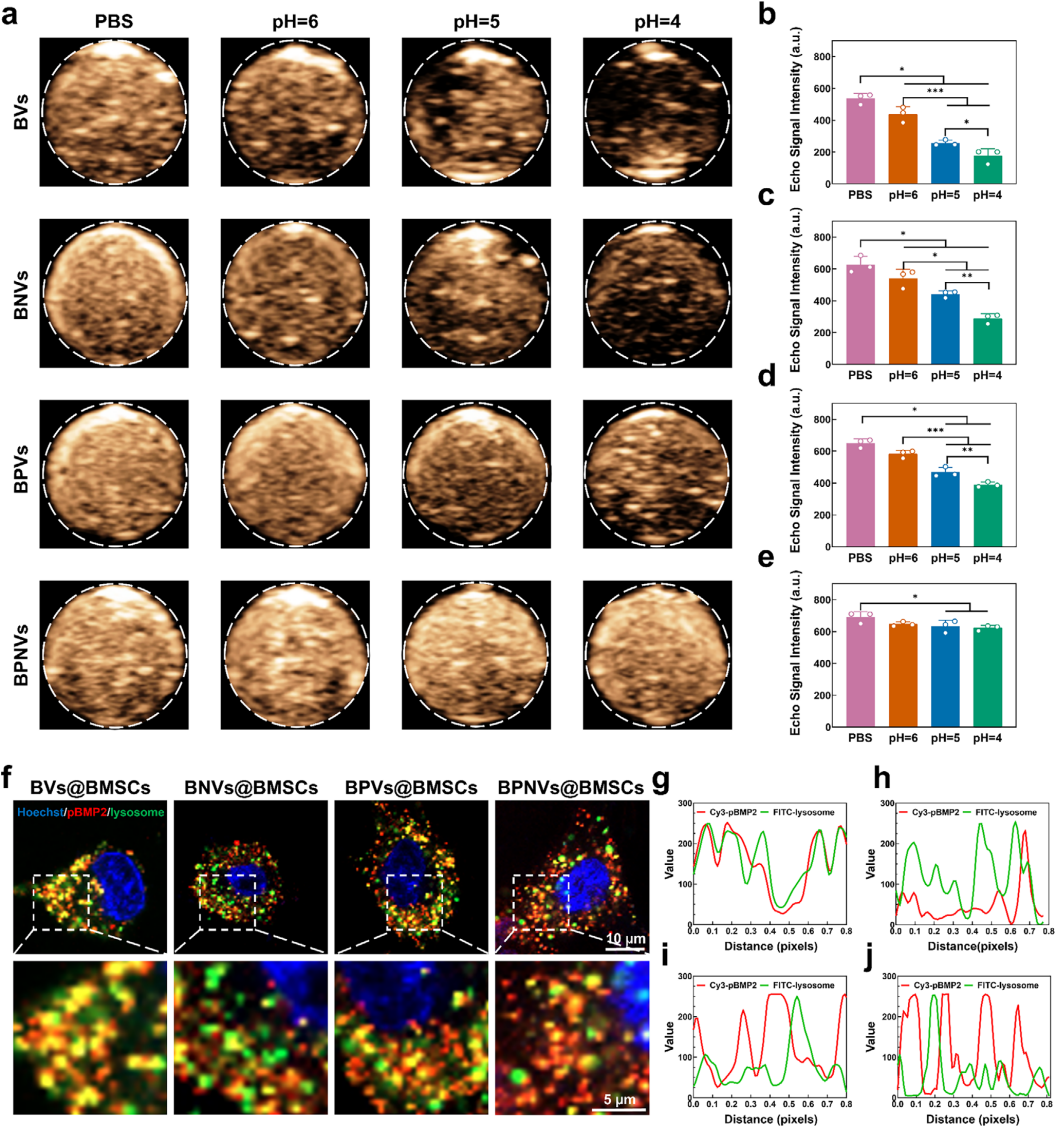
The lysosomal escape of BPNVs. a–e) In vitro contrast‐enhanced ultrasound images (a) and echo signal intensity of the agar phantom embedded with BVs (b), BNVs (c), BPVs (d), or BPNVs (e) in solutions with varied pH values (*n*  =  3). f) After co‐incubating BVs, BNVs, BPVs, and BPNVs with BMSCs for 8 h, the colocalization of pBMP2 and lysosome was observed by confocal microscope. g–j) Colocalization analysis of pBMP2 and lysosome in BMSCs of BVs@BMSCs (g), BNVs@BMSCs (h), BPVs@BMSCs (i), and BPNVs@BMSCs (j) group. ^*^ *p* < 0.5, ^**^ *p* < 0.01, ^***^ *p* < 0.001 by one‐way ANOVA with Bonferroni correction.

Most endocytic routes of nanomaterial cell uptake converge upon the lysosome.^[^ ^29^ ^]^ Considering that lysosomes contain acidic hydrolases capable of digesting proteins, nucleic acids, polysaccharides, and other biomolecules, the acid resistance of BPNVs is particularly important.^[^ ^30^ ^]^ Several particles have been mixed with hydrochloric acid to investigate acid tolerance. In the acid‐base titration experiment, the BPNVs group both with PEI and NLS exhibited the slowest pH decline, indicating the best buffering capacity, which was mostly attributed to PEI and synergistically to NLS (Figure S8, Supporting Information). As pH fell, the echo signal intensity of the BVs group (pBMP2 + GVs) significantly decreased. Nevertheless, the echo intensity in the BPNVs group exhibited minimal variation (**Figure** **3**a–e). This suggests that the modification with NLS and PEI enhances the stability and acid resistance of GVs. Fluorescence colocalization analysis showed that BPNVs were mainly located in the cytoplasm following phagocytosis by BMSCs, with minimal yellow fluorescence. However, the vast majority of BVs are situated within lysosomes and exhibit an amount of yellow fluorescence. Approximately half of BNVs (pBMP2 + NLS + GVs) and BPVs (pBMP2 + PEI + GVs) were situated in lysosomes, and yellow fluorescence was relatively reduced (Figure 3f–j). The colocalization coefficients (Pearson's *r*‐value) for pBMP2 and lysosomes in BVs@BMSCs, BNVs@BMSCs, BPVs@BMSCs, and BPNVs@BMSCs groups were respectively 0.8 ± 0.02, 0.7 ± 0.02, 0.54 ± 0.05, and 0.15 ± 0.08. It further confirmed that BPNVs could escape from lysosomes after entering BMSCs (Figure S9, Supporting Information).^[^ ^31^ ^]^ Furthermore, the TEM image revealed that BPNVs adhered to the lysosomal membrane, resulting in the pore formation. BPNVs escaped from the lysosomal membrane into the cytoplasm through the above pore (Figure S10, Supporting Information). It further confirmed the lysosomal escape function of BPNVs from a morphological perspective. This lysosomal escape capability and acid resistance of BPNVs may benefit from the modification of NLS and PEI. NLS contains a series of basic, positively charged amino acid sequences to condense DNA, which protects the nucleic acids from degradation.^[^ ^32^ ^]^ Besides, PEI's buffering capacity at low pH is crucial to the “proton‐sponge” hypothesis. The amino acids of PEI bind a large number of protons in the lysosome, resulting in the influx of Cl^−^ and water, which leads to the rupture of the lysosomal membrane by osmotic forces and the release of BPNVs.^[^ ^33^ ^]^

### 2.3 Intracellular Cavitation for the Enhancement of Nuclear Translocation

**Figure 4:**
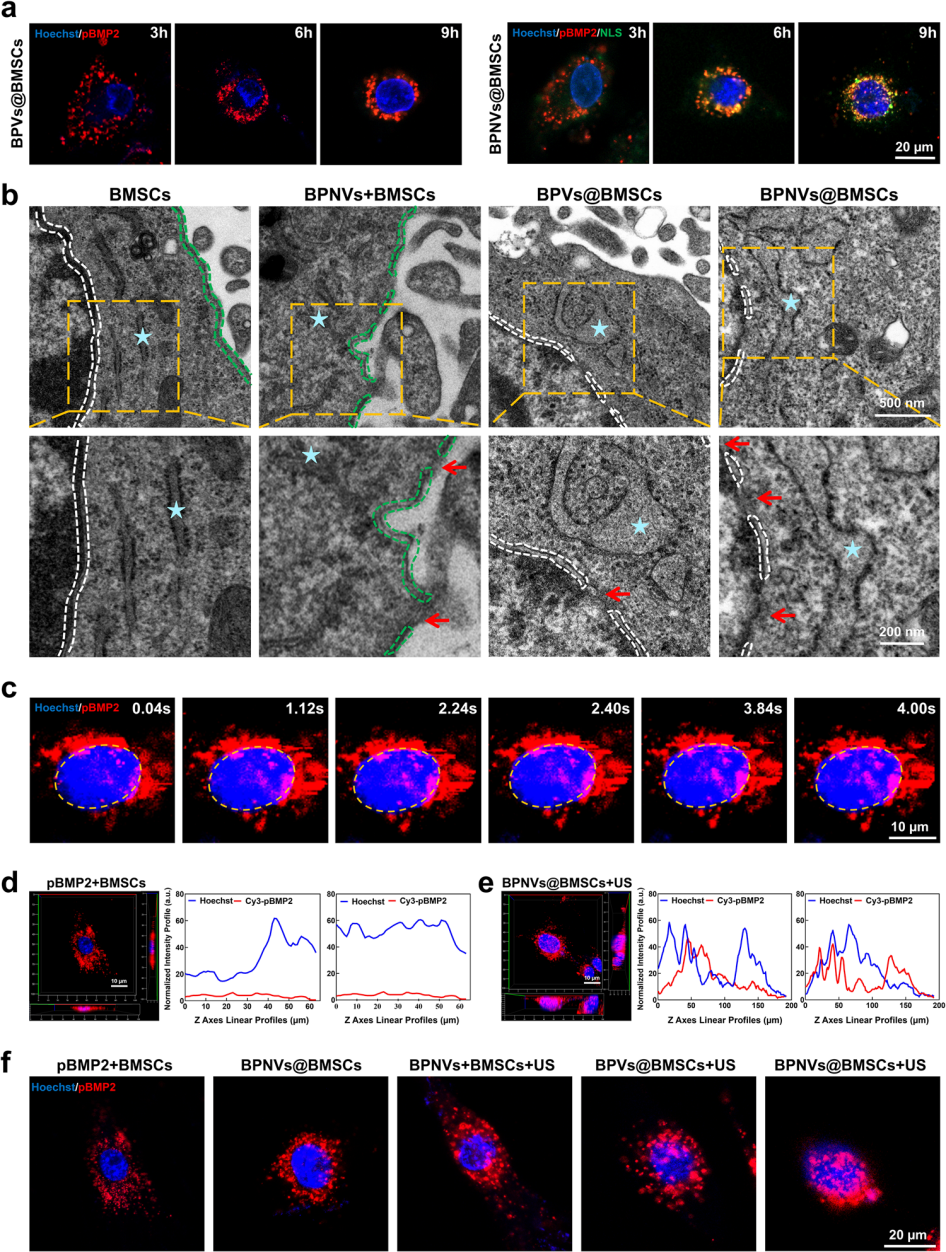
Active nuclear‐targeting effect of BPNVs and nuclear gene delivery by intracellular cavitation. a) Confocal microscopy images of BPVs or BPNVs co‐incubated with BMSCs for 3, 6, and 9 h. b) TEM images of BMSCs, BPNVs + BMSCs, BPVs@BMSCs, and BPNVs@BMSCs obtained immediately after LIPUS sonication. White dotted line: nuclear membrane, green dotted line: cell membrane, blue stars: endoplasmic reticulum, red arrow: destructed membrane. c) Confocal microscopy images of BPNVs@BMSCs after irradiation by LIPUS. White dotted line: nucleus membrane. d,e) The normalized intensity distribution of Cy3‐pBMP2 (red) and Hoechst (blue) fluorescence signals in the XZ and YZ planes. f) Confocal microscopy images of different groups 48 h after or without ultrasonic transfection.

It is reported that NLS can be recognized in the cytoplasm by importin‐α and importin‐β, subsequently activating the nuclear import machinery for enhanced nuclear translocation following cellular uptake.^[^ ^34^ ^]^ To further confirm the nuclear targeting capability of NLS, the distance between pBMP2 and the nuclear membrane was measured. The results indicated that the pBMP2 in the BPVs@BMSCs group was located further from the nucleus than the BPNVs@BMSCs group at the same time. Moreover, the pBMP2 in the BPNVs@BMSCs group exhibited an earlier nuclear translocation, demonstrating the superior active nuclear‐targeting efficacy of NLS (**Figure** **4**a; Figure S11, Supporting Information). The reduced distance between GVs and the nuclear membrane may enhance cavitation, decrease the required acoustic power, and minimize cellular damage.^[^ ^35^ ^]^ LIPUS was used to examine extracellular and intracellular cavitation. As Figure 4b and Figure S12 (Supporting Information) shown, the cell membrane of BPNVs+BMSCs group (GVs were outside of BMSCs) developed three ports with maximum diameters of 128 nm, but the nuclear membrane remained unaffected, and the rough endoplasmic reticulum did not expand. However, the nuclear membrane of BPVs@BMSCs and BPNVs@BMSCs groups (GVs were inside of BMSCs) formed two and three reversible pores with maximum diameters of 170 and 290 nm, respectively (the typical nuclear pore size is ≈30 nm), along with significantly expanded rough endoplasmic reticulum, indicating protein production. These pores disappeared within 8 h, suggesting that cavitation pores are reversible. Furthermore, the rough endoplasmic reticulum in the BPNVs@BMSCs groups remained enlarged, indicating that protein synthesis was still happening.^[^ ^36^ ^]^ The results indicated that the intracellular cavitation could be induced by LIPUS with intracellular cavitation nuclei, which notably enhanced the permeability of the nuclear membrane in comparison with the extracellular cavitation of traditional UTMD. Because of the active nuclear‐targeting efficacy of NLS, more cavitation nuclei were delivered around the nucleus, leading to larger and more abundant pores. The confocal microscopy images and fluorescence colocalization analysis also further confirmed that the intracellular cavitation facilitates the rapid entry of pBMP2 into the nucleus, whereas pBMP2 in the BPNVs@BMSCs group without ultrasonic irradiation hardly entered the nucleus (Figure 4c–e; Figure S13, Supporting Information). 48 h post‐transfection with optimized ultrasound parameters, a greater quantity of pBMP2 entered the nucleus in the intracellular cavitation groups (BPVs@BMSCs + US, BPNVs@BMSCs + US) compared to the extracellular cavitation group (BPNVs + BMSCs + US) (Figure 4f; Figure S14, Supporting Information). Quantitative analysis demonstrated that the targeted intracellular cavitation group (BPNVs@BMSCs + US) exhibited a nuclear translocation efficiency of 284.7% and 99.3% much higher than the extracellular cavitation group (BPNVs + BMSCs + US) and the non‐targeted intracellular cavitation group (BPVs@BMSCs + US), respectively (Figure S15, Supporting Information). These results indicated that the ultrasound‐mediated biotransfection of engineered BMSCs could deliver more cavitation nuclei around the nuclear membrane, while the intracellular cavitation could enhance nuclear translocation by directly improving the permeability of the nuclear membrane.

### 2.4 Transcription and Translation of pBMP2 Improved by Intracellular Cavitation

**Figure 5:**
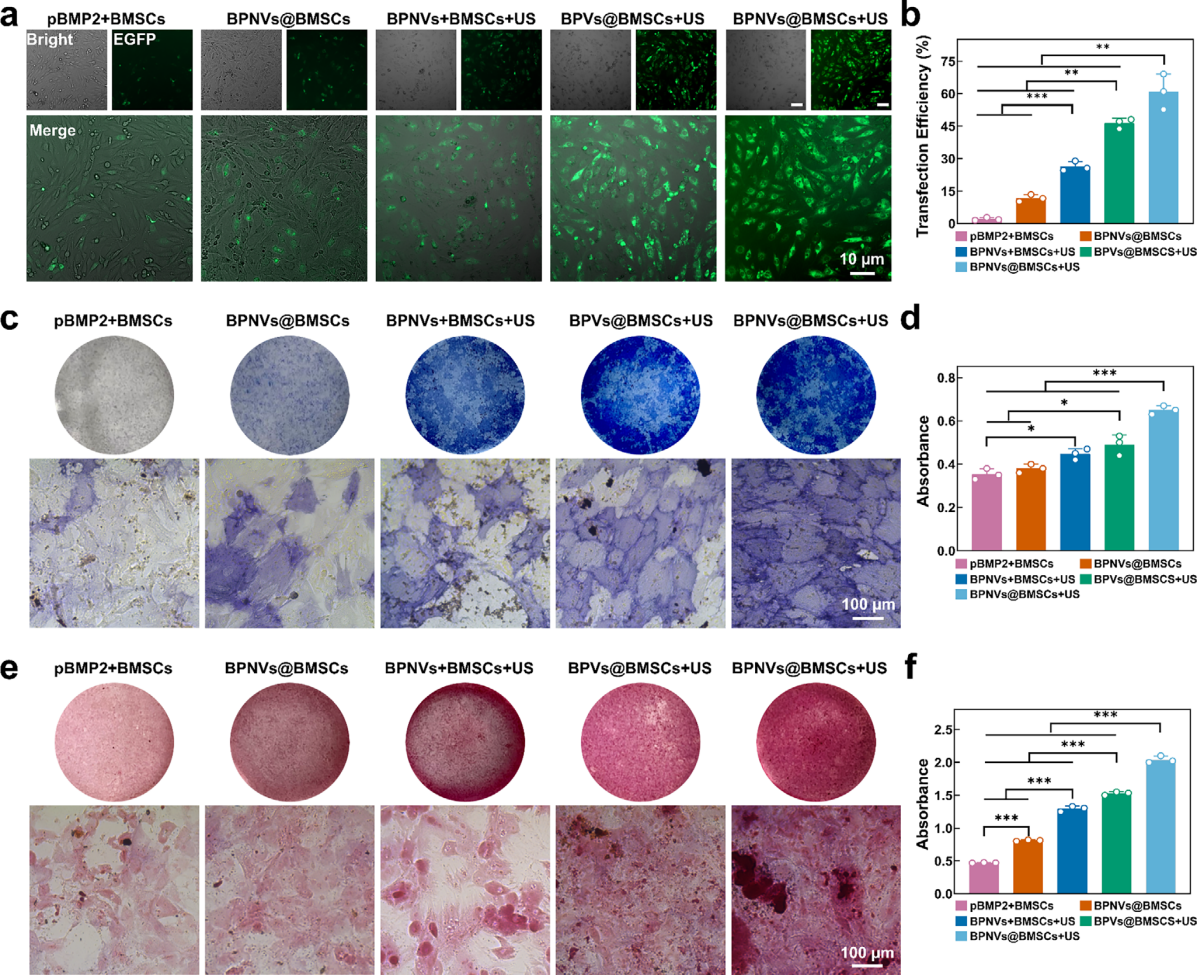
Transcription and translation of pBMP2 improved by intracellular cavitation. a,b) Expression of enhanced green fluorescent protein gene (green) (a) and quantification analysis of transfection efficiency by flow cytometry (b) in pBMP2 + BMSCs, BPNVs@BMSCs, BPNVs + BMSCs + US, BPVs@BMSCs + US, BPNVs@BMSCs + US groups (*n*  =  3). c,d) After 14 days in osteogenic differentiation medium, the above groups' early calcium nodule formation stained with alkaline phosphatase (blue) was quantified (c) and qualitatively (d) analyzed (*n*  =  3). e,f) After 21 days in osteogenic differentiation medium, the above groups' late mineralized nodule formation stained with alizarin red (red) was quantified (e) and qualitatively (f) analyzed (*n*  =  3). ^*^ *p* < 0.5, ^**^ *p* < 0.01, ^***^ *p* < 0.001 by one‐way ANOVA with Bonferroni correction.

We qualitatively and quantitatively examined the gene transfection efficiency across several groups to ascertain the influence of intracellular cavitation. The transfection efficiencies of the pBMP2 + BMSCs, BPNVs@BMSCs, BPNVs + BMSCs + US, BPVs@BMSCs + US, and BPNVs@BMSCs + US group were respectively 2.0 ± 0.6%, 11.7 ± 1.6%, 26.3 ± 2.2%, 46.3 ± 2.2%, and 60.9 ± 8.2%. The transfection efficiency of the targeted intracellular cavitation group (BPNVs@BMSCs + US) markedly increased by 131.6% and 31.5% compared to the extracellular cavitation group (BPNVs + BMSCs + US) and the non‐targeted intracellular cavitation group (BPVs@BMSCs + US), with a similar trend of the plasmid nuclear translocation efficiency (**Figure** **5**a,b). According to qPCR results, BMP2 mRNA expression in the targeted intracellular cavitation group rose by 117.5% and 70.1% compared to the extracellular cavitation and non‐targeted intracellular cavitation groups (Table S1, Figure S16, Supporting Information). In addition, Elisa analysis revealed sustained BMP2 production in BPNVs@BMSCs + US group for 21 days (Figure S17, Supporting Information). The growth of osteoblasts encompasses three stages: cell differentiation and proliferation, matrix synthesis, and matrix calcification.^[^ ^37^ ^]^ We utilized alkaline phosphatase and alizarin red to signify the early and later phases of osteogenesis.^[^ ^38^ ^]^ In comparison to the pBMP2+BMSCs group, the BPNVs@BMSCs+US group had much earlier osteogenesis, with more pronounced mineralized nodule development than the other four groups (Figure S18, Supporting Information). Significantly, these nodules demonstrated enhanced matrix mineralization capacity and osteogenic efficacy (Figure 5c–f; Figure S19, Supporting Information). Taken together, the ultrasound‐mediated biotransfection of engineered BMSCs can efficiently promote the differentiation of BMSCs into osteoblasts through the transcription and translation of BMP2 protein.

### 2.5 Ultrasound‐Mediated Biotransfection of Engineered BMSCs for Bone Healing Efficacy

**Figure 6:**
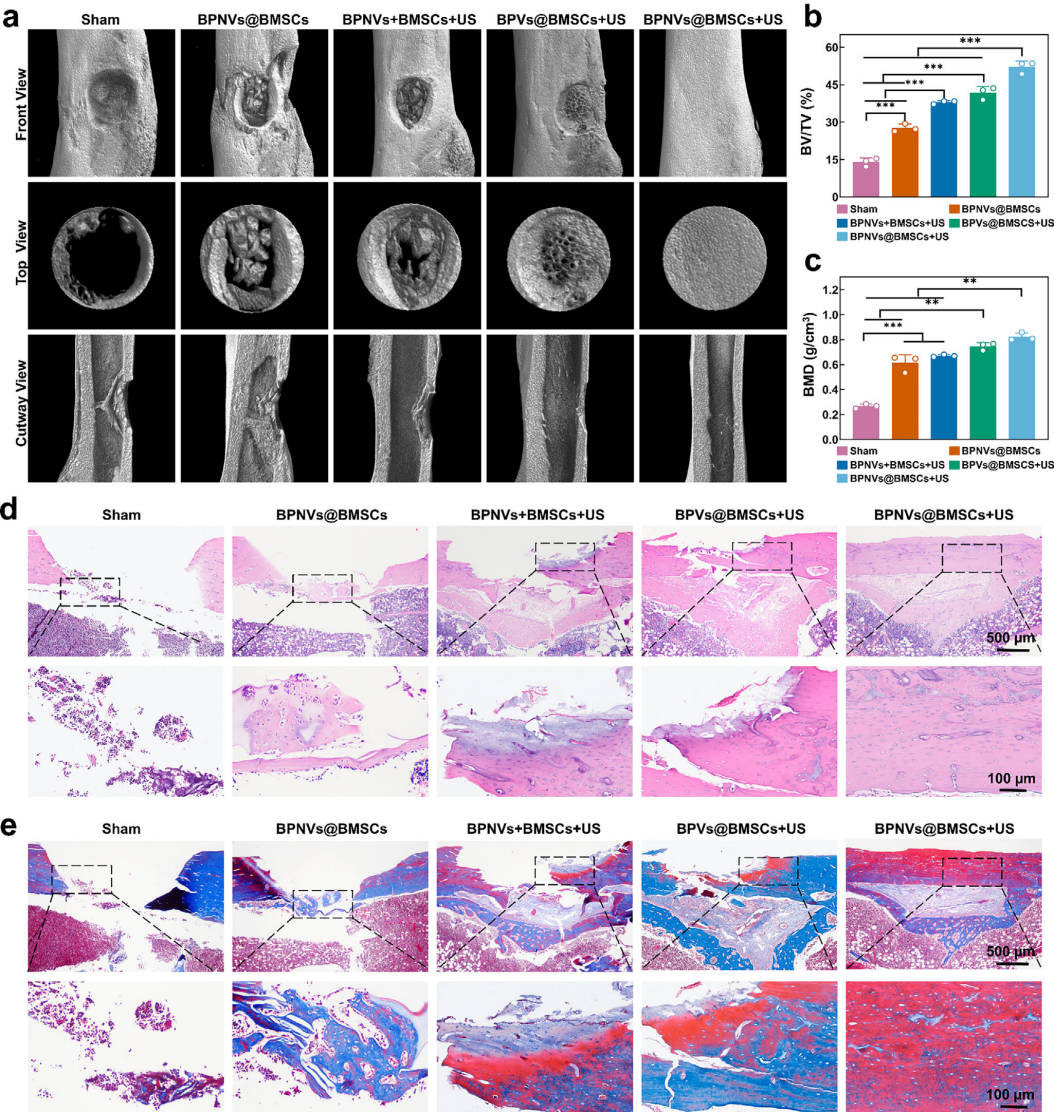
Ultrasound‐mediated biotransfection of engineered BMSCs for bone healing. a) The 3D reconstruction images of femoral defects in each group from the frontal, top, and cutaway positions. b,c) The bone‐related parameters based on the micro‐CT analysis, embracing BV/TV (b) and BMD (*n*  =  3). d,e) Assessment of regenerated bone in various groups using H&E staining (d) and Masson staining (e) for histological analysis. Blue represents newly formed bone tissue, while red represents mature bone tissue. ^**^ *p* < 0.01, ^***^ *p* < 0.001 by one‐way ANOVA with Bonferroni correction.

**Figure 7:**
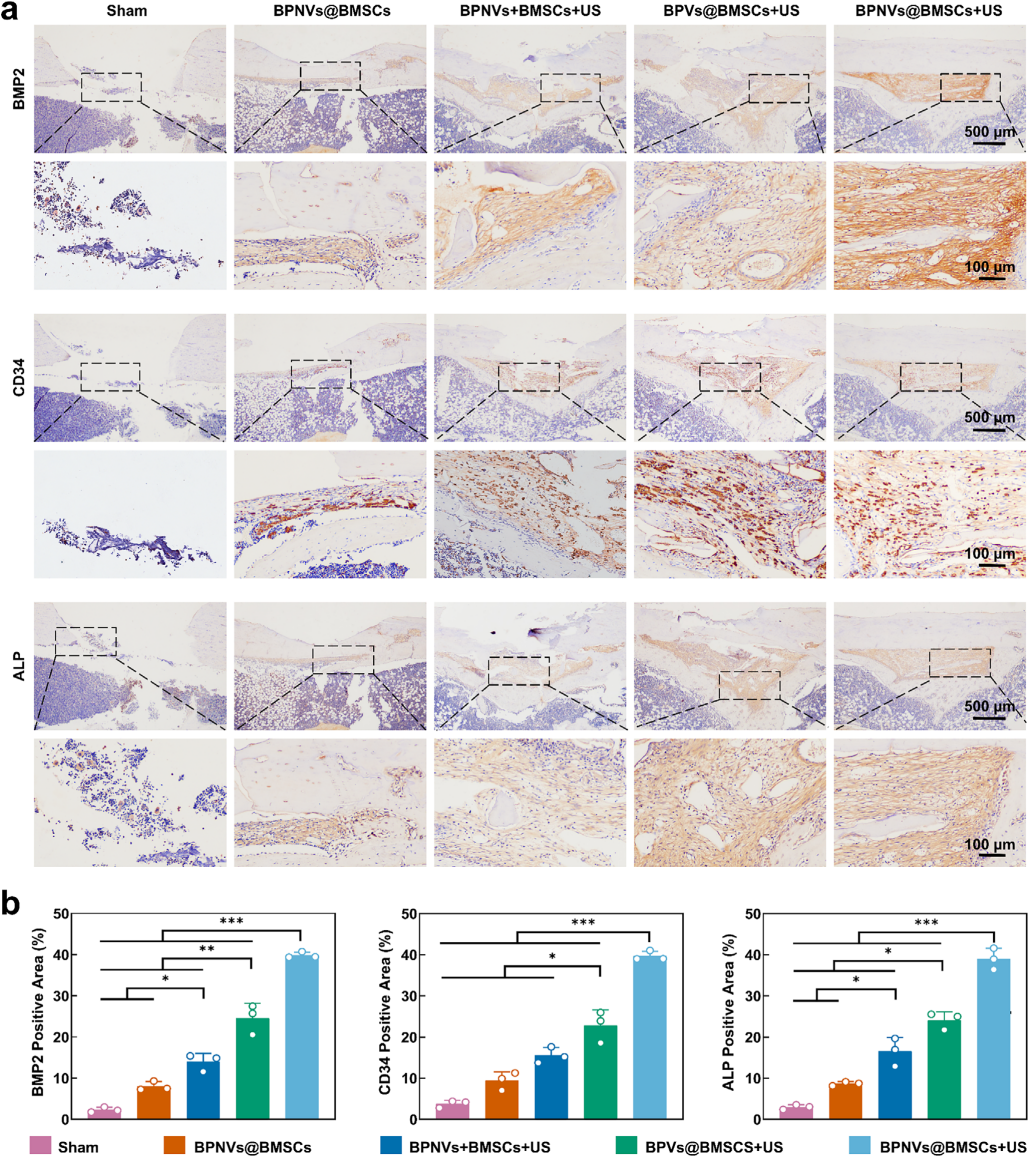
Effects of engineered BMSCs on the expression of related proteins in femoral defect tissues. a) The representative microscopy images of immunohistochemical staining of osteogenic‐related proteins in femoral defect tissues. b) Quantitative analysis of positive stained rate of BMP2, cluster of differentiation 34 (CD34), and alkaline phosphatase (ALP) proteins in the above groups (*n*  =  3). ^**^ *p* < 0.01, ^***^ *p* < 0.001 by one‐way ANOVA with Bonferroni correction.

Engineered BMSCs were injected with gelatin methacryloyl (GelMA) hydrogel in situ into the bone defect of rats to further investigate the bone healing effects. 21 days after injection, BPNVs@BMSCs remained stably located at the bone defect site (Figure S20 and S21, Supporting Information). After finishing the corresponding treatments for 4 weeks, the micro‐computed tomography (micro‐CT) results indicated that there is massive new bone formation in the BPVs@BMSCs+US group compared to the Sham group, BPNVs@BMSCs group, and BPNVs+BMSCs+US group. However, there were still certain gaps present in the BPVs@BMSCs+US group, whereas the bone defects in the BPNVs@BMSCs+US group were almost completely repaired (**Figure** **6**a; Figure S22a, Supporting Information). Moreover, based on the micro‐CT analysis, the bone‐related parameters in each group, including bone mineral density (BMD), bone volume fraction (BV/TV), bone surface area density (BS/BV), trabecular thickness (Tb. Th), trabecular separation (Tb. Sp), and trabecular number (Tb. N) presented the same altered tendency (Figure 6b,c; Figure S22b, Supporting Information). The Sham, BPNVs@BMSCs, BPNVs+BMSCs+US and BPVs@BMSCs+US groups all exhibited new bone formation, but the efficacy of bone regeneration remained unsatisfactory. In the BPNVs@BMSCs+US group, the bone defect was entirely filled with newly formed bone. This led to the evident development of mature bone (Figure 6d,e; Figure S23, Supporting Information). It indicated that ultrasound‐mediated biotransfection of engineered BMSCs accelerated the repair of bone defects. Based on the histological staining results demonstrating the therapeutic effects of engineered BMSCs on bone defects, we subsequently assessed the expression of proteins associated with the regeneration of vascular and bone tissues to further explore the molecular effects of engineered BMSCs. As shown in **Figure** **7**, a significant increase in the expression levels of osteogenic‐related proteins in the BPNVs@BMSCs + US groups compared to the other groups.^[^ ^39^ ^]^ This implied that ultrasound‐mediated biotransfection of engineered BMSCs improved the osteogenic microenvironment in bone defects and completely repaired bone defects with more mature regenerated bone. Furthermore, the hematoxylin and eosin (H&E) staining sections of tissues (heart, liver, spleen, lung, kidney) exhibited no obvious lesions (absence of necrosis, edema, inflammatory infiltration, or hyperplasia) across the various groups, providing evidence that the ultrasound‐mediated biotransfection of engineered BMSCs was a relatively safe therapy for bone defects (Figure S24, Supporting Information).

## 3 Conclusion

This study presented the advancement of ultrasound‐mediated biotransfection of engineered BMSCs employing the intracellular cavitation enabled by LIPUS. It exhibited remarkable lysosome escape capacity and excellent nuclear targeting ability. LIPUS irradiation induced the intracellular cavitation, enhancing nuclear membrane permeability and facilitating the nuclear delivery of pBMP2, thereby respectively augmenting the plasmid nuclear translocation efficiency and gene transfection efficiency by 284.7% and 131.6% in comparison to the extracellular cavitation and non‐targeted intracellular cavitation groups. Through the production and release of BMP2, engineered BMSCs significantly promoted bone regeneration by improving the osteogenic microenvironment of bone defects. This research offered a more secure, efficient, and regulated approach to BMSCs gene therapy for bone defects. The novel ultrasound‐mediated biotransfection of engineered BMSCs inspired the advancement of gene transfection methods, particularly for challenging cell lines that are difficult to transfect.

## 4 Experimental Section

### Acid Resistant Capacity

To evaluate the acid buffering capacity of transfection complexes, solutions with varying pH levels were prepared to simulate the physiological and lysosomal acidic environments in vitro. BVs, BNVs, BPVs, and BPNVs were dissolved in the above solutions, subsequently imaged and analyzed by ultrasound (Aplio i800 with i18LX5, Canon, Japan). Furthermore, the solutions from various experimental groups were titrated using a 0.1 m standard hydrochloric acid (HCl) solution. The alterations in pH levels of the solutions throughout the titration process, together with the volume of HCl administered, were recorded.

### Lysosomal Escape Analysis

Lysotracker green was utilized to label lysosomes, while an intracellular nucleic acid localization kit was employed to label plasmids. Nuclei were stained with Hoechst 33342. BVs, BNVs, BPVs, and BPNVs were incubated with BMSCs for 8 h, respectively. The spatial relationship between plasmids and lysosomes was observed by confocal microscopy.

### Changes in Nuclear Membrane Permeability Induced by Intracellular Cavitation

Following ultrasound irradiation of BPNVs@BMSCs, time‐series imaging was performed using confocal microscopy to dynamically monitor the localization of plasmids (labeled with an intracellular nucleic acid localization kit) relative to nuclei (stained with Hoechst 33342). The number of plasmids was quantified using ImageJ. 3D confocal imaging was employed to assess changes in plasmid numbers within the nucleus post‐irradiation. TEM (JEM 1400, JEOL, Japan) was further utilized to investigate nuclear membrane pores and morphological changes of the endoplasmic reticulum in different groups.

### Assessment of In Vitro Gene Transfection Efficiency

Using the optimized parameters for sonication, transfection efficiency was evaluated through fluorescence microscopy and flow cytometry. The subcellular localization of plasmids was observed by confocal microscopy (LSM800, Zeiss, Germany), and ImageJ was employed to calculate the nuclear translocation efficiency.

### Establishment of Bone Defect Rat Model and Treatment Protocol

All animal experiments were performed in accordance with the institutional guidelines and approved by the Ethics Committee of Guangdong Second Provincial General Hospital (Approval No. 2023‐DW‐KZ‐006‐01). Anesthesia was induced via intraperitoneal injection of 3% sodium pentobarbital (0.3 mL kg^−1^), followed by exposure of the femur through muscle dissection. A 3.2 mm bone defect was created in the upper femoral segment using an orthopedic electric drill. Cells from each group were suspended in 30 µL of GelMA hydrogel and injected into the bone defect site, then immediately irradiated with light at a wavelength of 405 nm to facilitate rapid photopolymerization. Postoperatively, penicillin was administered intramuscularly at a dose of 80 000 U/100 g. After 4 weeks, all rats were euthanized, and the femurs were extracted for subsequent analysis.

### Statistical Analysis

The data of this study were statistically analyzed using SPSS 25.0 software (IBM, Armonk, NY, USA). Quantitative experimental data were expressed as mean ± standard deviation. Student's *t*‐test was used for comparison between two groups, while one‐way ANOVA was used for comparison between multiple groups. A *p*‐value of less than 0.05 (*p* < 0.05) was considered statistically significant.

## Conflict of Interest

The authors declare no conflict of interest.

## Supporting information



Supporting Information

## Data Availability

The data that support the findings of this study are available from the corresponding author upon reasonable request.
