# Supplementary material for: Ultrasound‐Mediated Biotransfection of Engineered Bone Marrow Mesenchymal Stem Cells in Treated Bone Defects through Intracellular Cavitation
Source: Adv Sci (Weinh). 2025 Jul 30;12(40):e03196. doi: 10.1002/advs.202503196 (PMC12561189; doi:10.1002/advs.202503196)
Supplement: Supplementary file 1 — Supporting Information [file ADVS-12-e03196-s001.docx]

Supporting Information

Ultrasound-Mediated Biotransfection of Engineered Bone Marrow Mesenchymal Stem Cells in Treated Bone Defects Through Intracellular Cavitation

Zhili Xu, Huijuan Xin, Yu Wang, Renhao Xu, Yanni He, Meijun Zhou, Zhengqiang Yuan*, Hongmei Liu*

Z. Xu, H. Xin, Y. Wang, R. Xu, Y. He, M. Zhou, H. Liu

Department of Ultrasound

Institute of Ultrasound in Musculoskeletal Sports Medicine

The Affiliated Guangdong Second Provincial General Hospital of Jinan University

Guangzhou, 510317, China
E-mail: liuhm@gd2h.org.cn

Z. Yuan
School of Biomedical and Pharmaceutical Sciences

Guangdong University of Technology

Guangzhou, 510006, China

E-mail: yuanzq@gdut.edu.cn

Experimental Section

*Materials*: The nuclear localization signal (NLS; PKKKRKV) was purchased from Shanghai Science Peptide Biotechnology Co., Ltd. (China). Polyethyleneimine (PEI) and 1,1'-dioctadecyl-3,3,3',3'-tetramethylindocarbocyanine perchlorate (DiI) were obtained from Sigma-Aldrich (St. Louis, MO, USA). The pEF1α-BMP2(rat)-EGFP plasmid was procured from MiaoLing Plasmid Platform (China). The Cell Counting Kit-8 (CCK-8) was purchased from Dojindo (Kumamoto, Japan). The intracellular nucleic acid localization kit was sourced from Mirus Bio LLC (Madison, WI, USA). Enzyme-linked immunosorbent assay (ELISA) kits were purchased from Wuhan Huamei Biotech Co., Ltd. (China). Lyso-tracker, Hoechst 33342, tumor necrosis factor-alpha (TNF-α), and alkaline phosphatase (ALP) assay kits were obtained from Beyotime Biotechnology Co., Ltd. (China). RNA extraction kits were purchased from Vazyme Biotech Co., Ltd. (China). High-fidelity cDNA reverse transcription kits were purchased from Thermo Fisher Scientific (Waltham, MA, USA). Immunohistochemical antibodies used included bone morphogenetic protein 2 (BMP2; AF5163, Affinity Biosciences), cluster of differentiation 34 (CD34; ab81289, Abcam), and ALP (DF6225, Affinity Biosciences). Gelatin methacryloyl (GelMA) hydrogel was purchased from Engineering For Life Co., Ltd. (China).

*Characterization of Gas Vesicles (GVs):* The concentration of GVs was quantified by measuring the optical density at 500 nm using a multimode plate reader (Synergy 4, BioTek, USA). Morphological characteristics, particle size, and zeta potential were analyzed using a transmission electron microscope (TEM; JEM 1400, JEOL, Japan) and particle size analyzer (Zetasizer Nano ZS, Malvern Panalytical, UK).

*Preparation and Characterization of PEI/NLS-loaded GVs (PNVs):* To construct NLS-loaded GVs (NVs), NLS was incubated with GVs (OD_500_ = 1) for 20 min at varying doses (30, 60, 90, and 120 µg). Subsequently, unbound NLS was removed. PNVs were prepared by adding PEI at different doses (10, 20, 30, and 40 µg) to NVs (NLS = 90 µg), followed by the removal of unbound PEI through low-speed centrifugation. The morphological characteristics, particle size, and zeta potential of both NVs and PNVs were analyzed using TEM (JEM1400, JEOL, Japan) and particle size analyzer (Zetasizer Nano ZS, Malvern Panalytical, UK).

*Preparation and Characterization of Engineered Bone Marrow Mesenchymal Stem Cells (BPNVs@BMSCs):* To assess the gene loading capacity of PNVs, agarose gel electrophoresis was conducted. pBMP2/PEI/NLS-loaded GVs (BPNVs) were subsequently prepared by incubating PNVs with varying doses of PEI (2.5, 5, 10, 15, 20, and 25 µg) to 10 µg pBMP2. The resulting BPNVs were labeled with DiI and co-incubated with BMSCs for 3, 6, 9, and 12 h to form BPNVs@BMSCs. The optimal phagocytosis time was determined using flow cytometry (BD FACSCanto^TM^ II, Becton Dickinson) and fluorescence microscopy (Axio Observer Z1, Zeiss), with BMSCs serving as the control group. The morphology and subcellular localization of BPNVs within BMSCs were further examined using TEM and laser scanning confocal microscopy (LSM800, Zeiss, Germany).

*In vitro Biosafety Analysis:* BMSCs were extracted from the femurs of 4-week-old male rats. The cells were then incubated with different concentrations of GVs (OD_500_ = 0.25, 0.5, 1, and 1.5) for 9, 12, 24, 36, and 72 h, after which the optimal incubation concentrations were determined using a CCK-8 assay. Subsequently, BMSCs, GVs@BMSCs, and PNVs@BMSCs were cultured for 21 days in osteogenic, adipogenic, and chondrogenic induction medium to assess differentiation potential. Calcium deposits, lipid droplets, and chondrocytes were stained with Alizarin Red, Oil Red O, and Alcian Blue, respectively, to evaluate the differentiation outcomes.

*Inflammatory Chemotactic Capacity:* BMSCs or BPNVs*@*BMSCs (1×10^3^ cells/well) were seeded in the upper chamber of a Transwell plate (24-well insert, pore size 8 µm). Complete DMEM supplemented with or without A-α (20 µg/mL) was added to the lower chamber. After 12 h, the cells that migrated to the lower side of the transwell membranes were fixed and stained with 0.2% crystal violet. The number of migrated cells was quantified using a light microscope.

*Nuclear Targeting Effect:* The intracellular nucleic acid localization kit was utilized to label pBMP2, FITC to label NLS, and Hoechst 33342 to label the nucleus. BPVs and BPNVs were incubated with BMSCs for 3, 6, and 9 h, respectively. The position of the plasmid relative to the nucleus was observed by confocal microscopy.

*Optimization of Ultrasound-Mediated In Vitro Gene Transfection Parameters:* The ultrasonic system utilized in the study consisted of an arbitrary waveform generator (DG4162, Rigol, China), an RF power amplifier (LZY-22+, MINI, USA), and a single-element focused ultrasound transducer (20 mm diameter, 1.5 MHz, 50 mm focal length; Seyfert Corporation). The transducer was positioned beneath the well plate, and BPNVs@BMSCs were exposed to varying sound pressures (100, 110, 120, and 130 mVpp) for different durations (2, 2.5, 3, and 3.5 min). After transfection, the cells were cultured for 48 h, and the optimal sound pressure and irradiation time were determined through flow cytometry (FACS Caliber, Becton Dickinson, USA).

*Evaluation of In Vitro pBMP2 Expression:* Quantitative reverse transcription polymerase chain reaction (qRT-PCR) was utilized to assess the differential expression of the BMP2 gene across experimental groups. Total RNA was extracted using an RNA extraction kit, followed by cDNA synthesis utilizing a high-fidelity cDNA reverse transcription kit. Target-specific primer sequences were incorporated. The qRT-PCR analysis was conducted using the LightCycler® 96 System (Roche, Switzerland), with glyceraldehyde-3-phosphate dehydrogenase (GAPDH) serving as the internal reference gene. The relative expression levels of BMP2 were calculated using the 2^-ΔΔCt^ method, which quantifies target gene expression relative to GAPDH. Supernatants were collected at 4, 9, 13, 17, and 21 days after transfection, and BMP2 protein concentrations were quantified using an ELISA kit.

*Assessment of Osteogenic Potential:* At 48 h post-gene transfection, cells were cultured in osteogenic differentiation medium. On days 14 and 21, cells from each group were stained with alkaline phosphatase and alizarin red, respectively. Alkaline phosphatase activity was measured using an alkaline phosphatase assay kit, and calcium nodules were quantified using a 10% cetylpyridinium chloride solution. Enhanced green fluorescent protein (EGFP) expression and calcium nodules formation in the BPNVs@BMSCs group were assessed on days 3, 5, 7, and 14 using fluorescence microscopy.

*Evaluation of Bone Healing Efficacy:* Micro-computed tomography (Micro-CT; ZKKS-MCT-Sharp, Caskaishen, China) was used for three-dimensional reconstruction of the femoral defect site, utilising the following scanning parameters: beam energy of 60 kV, current of 100 μA, and a resolution of 18 μm. Quantitative analysis of bone formation was performed by assessing bone mineral density (BMD), bone volume fraction (BV/TV), bone surface area density (BS/BV), trabecular thickness (Tb. Th), trabecular separation (Tb. Sp), and trabecular number (Tb. N). Furthermore, post-imaging, bone tissue was collected for histological analysis, including hematoxylin and eosin (H&E) staining, Masson staining, and immunohistochemical staining for BMP2, CD34, and ALP.

Supplementary Results

**Table S1.** Primers Sequence.

| Primer Name | Primer Sequence (5’-3’) |
| --- | --- |
| GAPDH- Forward | CACggCAAgTTCAACggCACAg |
| GAPDH- Reverse | TggTgAAgACgCCAgTAgACTC |
| BMP2- Forward | gTTCTgTCCCTACTgATgAg |
| BMP2- Reverse | gTCCAATAgTCTggTCACAgg |


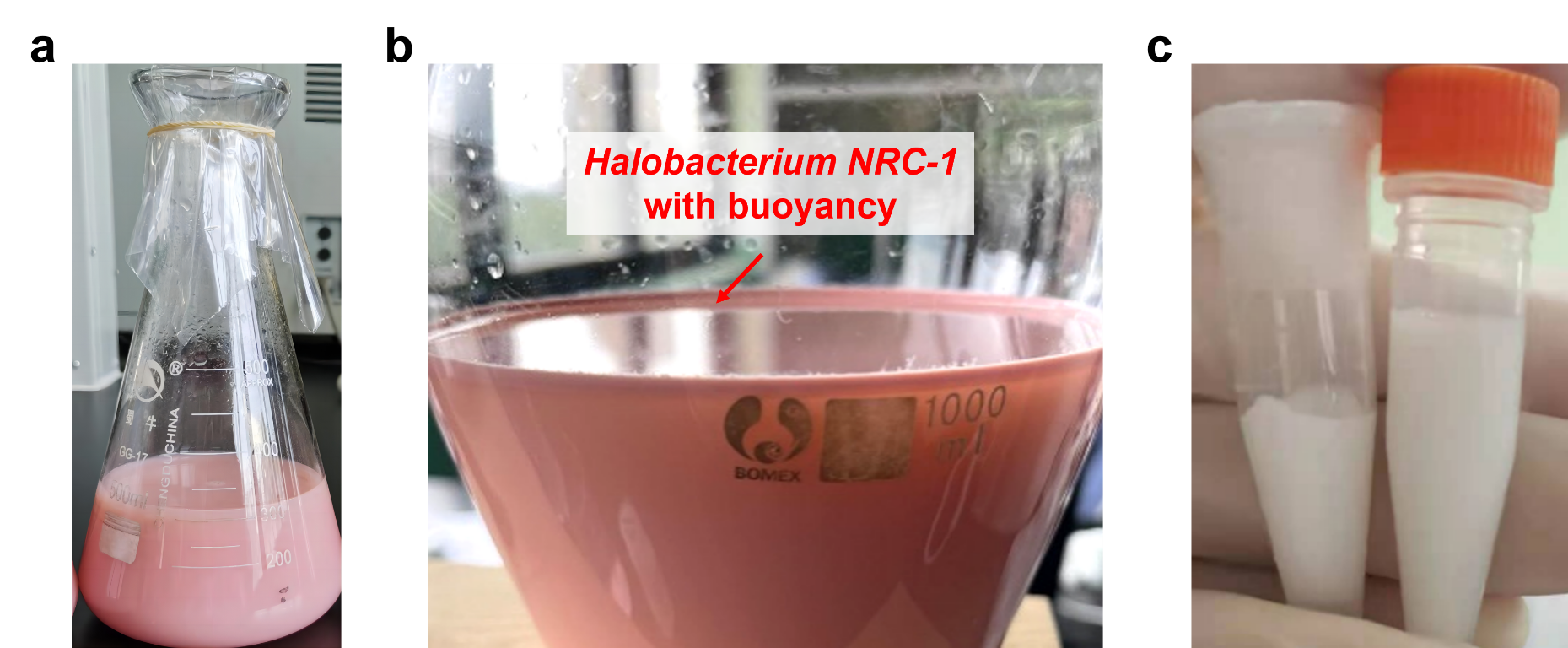


**Figure S1.** Isolation and purification of GVs. a) *Halobacterium NRC-1* of pink. b) A liquid separation funnel is used to extract buoyant bacteria. c) GVs were extracted and refined from bacteria, then suspended in PBS.


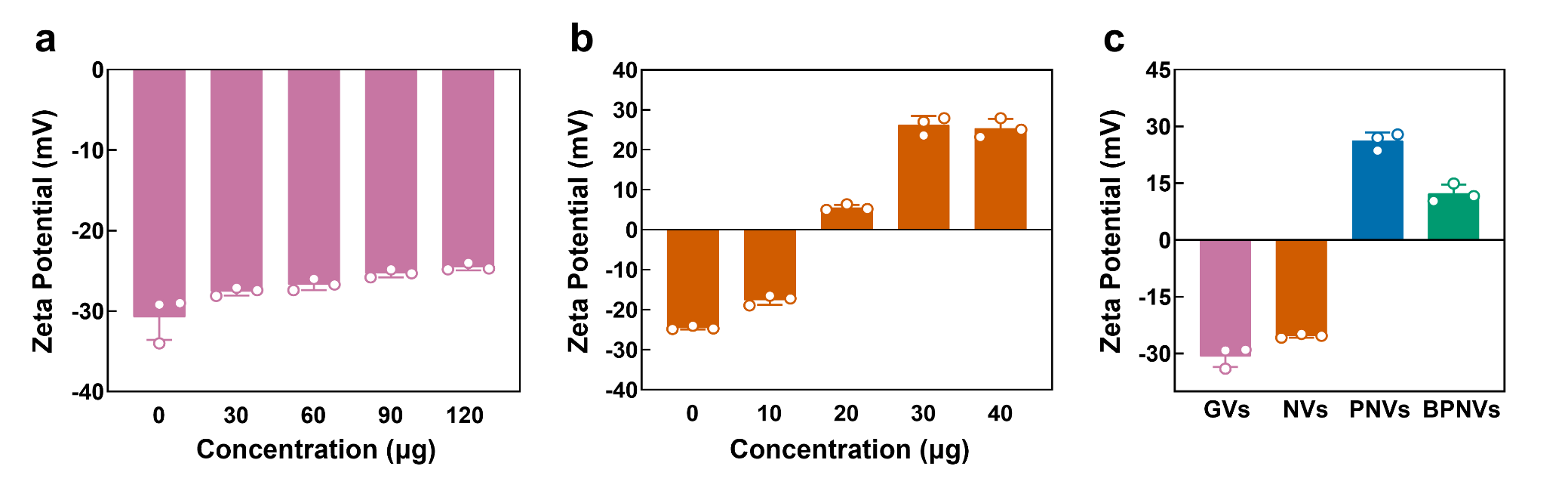


**Figure S2.** Zeta potential of different nanoparticles. a) The potential change diagram of NVs with different concentrations of NLS. b) The potential change diagram of PNVs with different concentrations of PEI. c) The zeta potential of GVs, NVs, PNVs and BPNVs (*n* = 3).


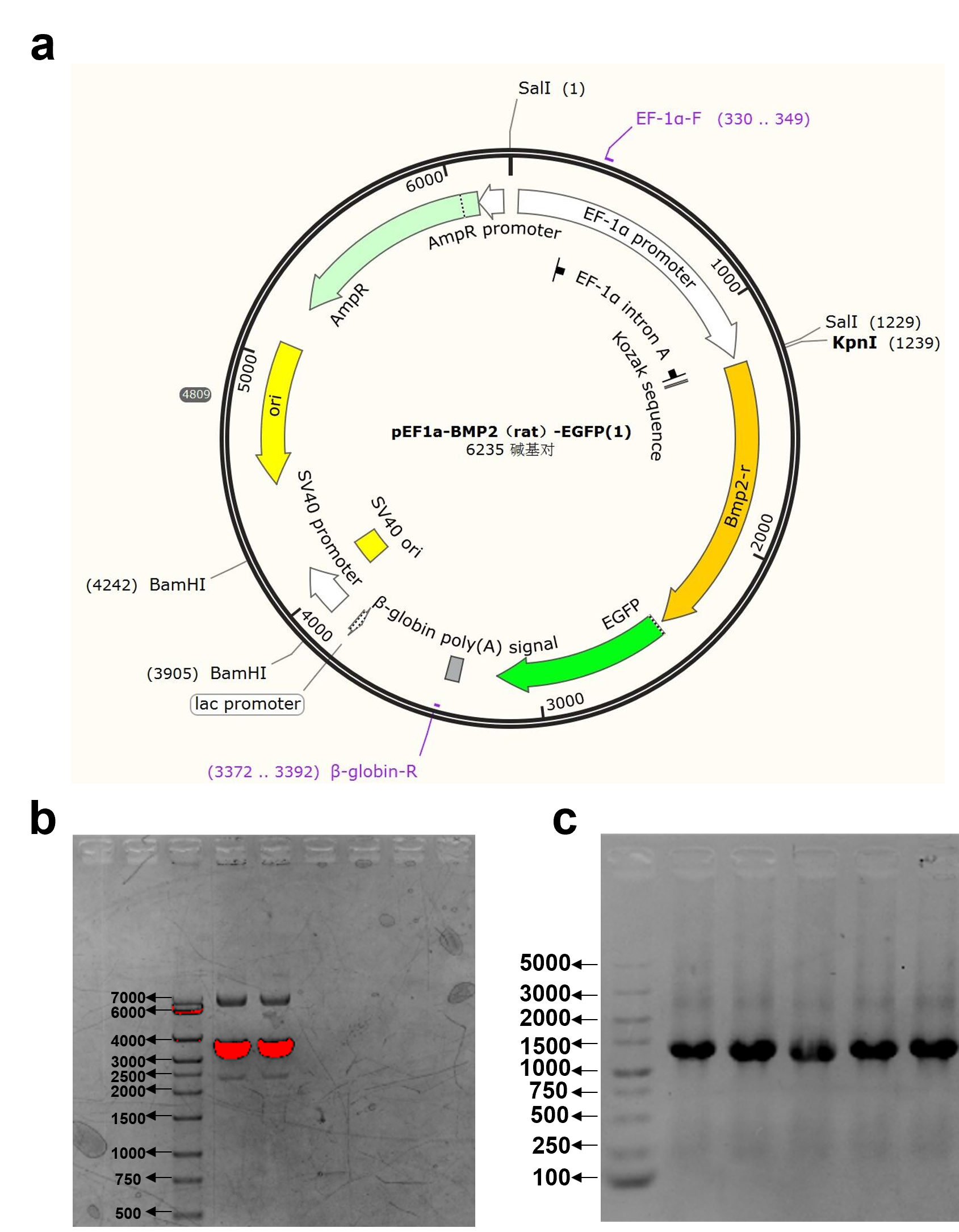


**Figure S3.** Characterization of pBMP2. a) Gene map of the pEF1a-BMP2 (rat)-EGFP. b) The plasmid was confirmed using agarose gel electrophoresis. c) The electrophoretic map indicated that the BMP2 gene fragment had a size of 1182 base pairs.


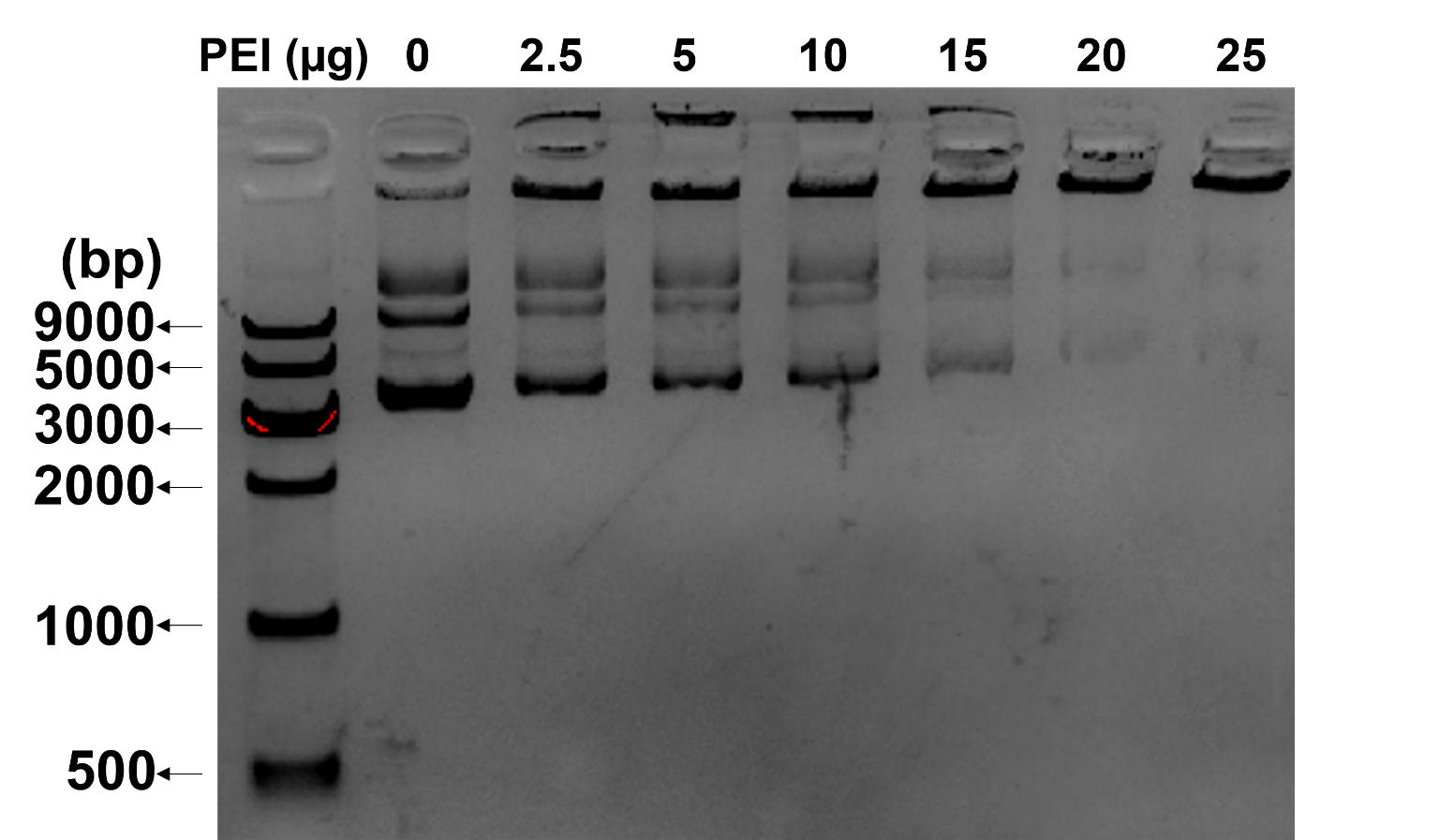


**Figure S4.** Agarose gel electrophoresis map of 10 μg PEI plasmid combined with BPNVs containing varying amounts of pBMP2.


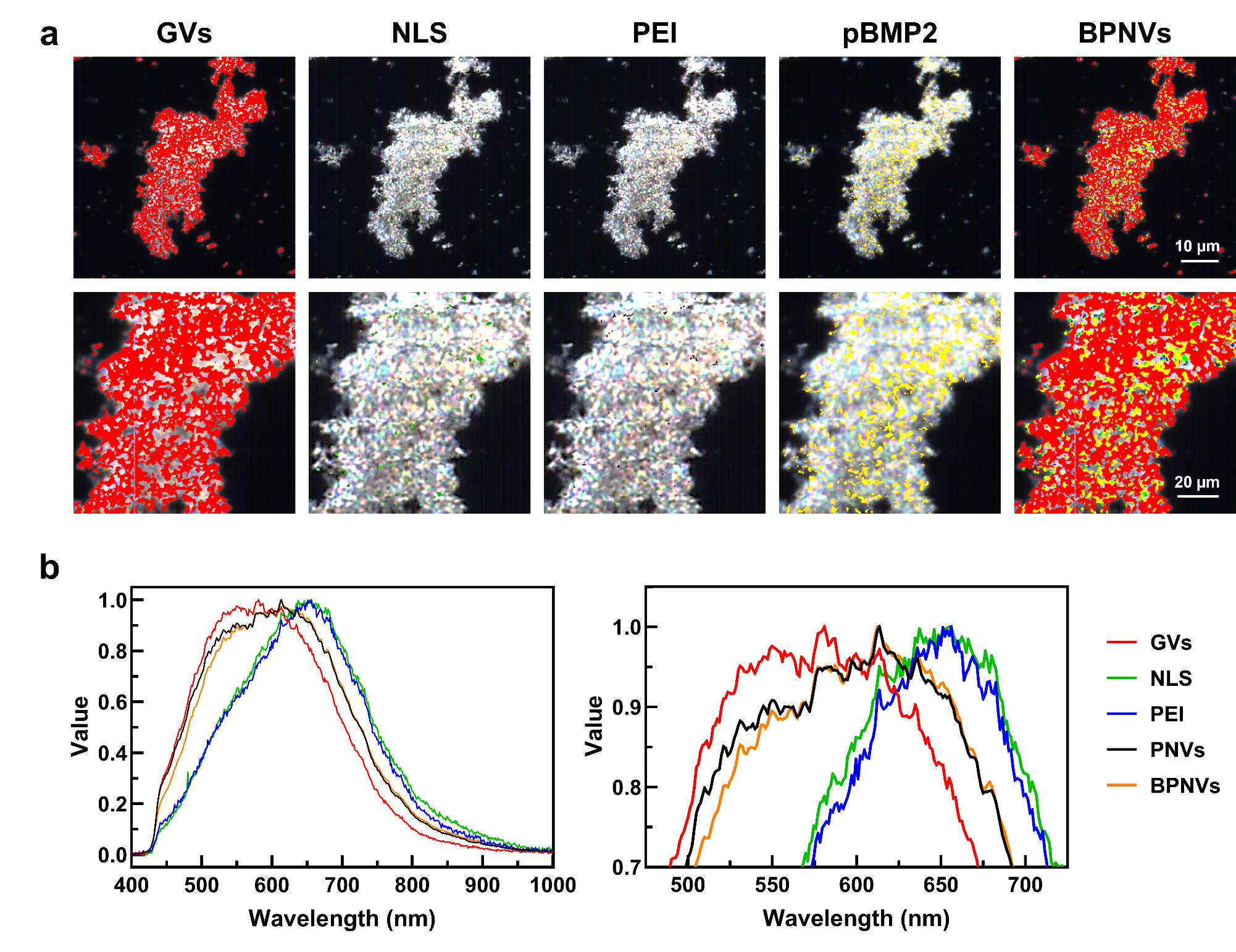


**Figure S5.** EDHM analysis of BPNVs. a) EDHM imaging of BPNVs. b**)** Hyperspectral signal curves generated for BPNVs. The right figure is a detailed enlarged view of the left figure.


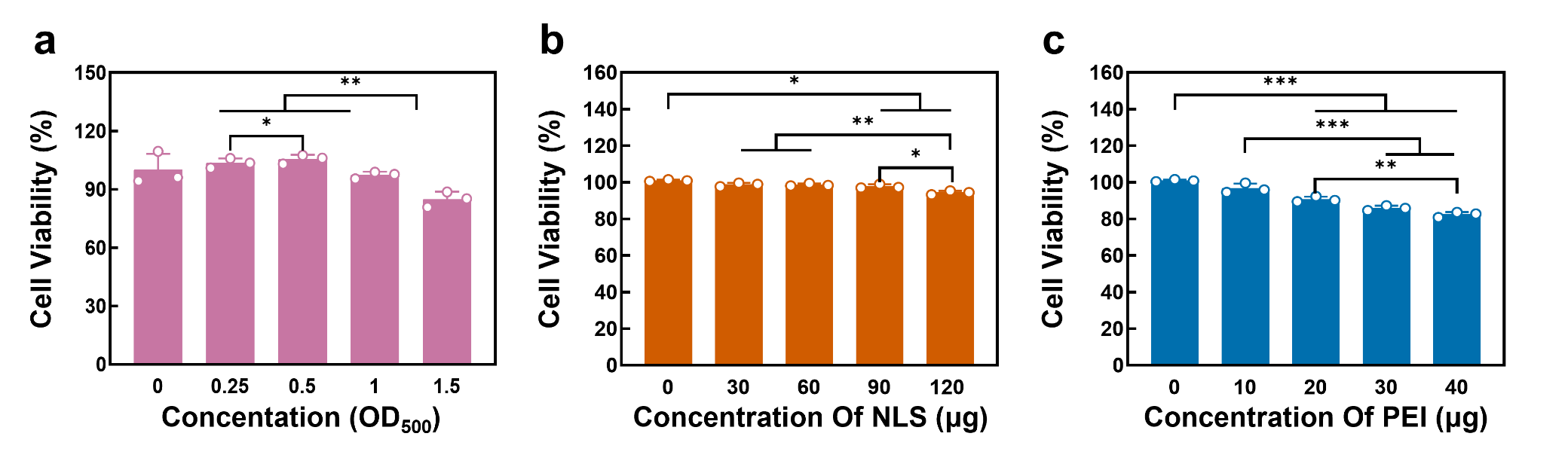


**Figure S6.** The biological safety of GVs, NLS, and PEI for BMSCs. a) Percentages of viable BMSCs with different concentrations of GVs. b) Percentages of viable BPNVs@BMSCs with varying concentrations of NLS. c) Percentages of viable BPNVs@BMSCs containing 20 μg PEI at 37 °C for 0, 10, 20, 30, 40 h (*n* = 3). **p* <0.5, ** *p* <0.01, ****p* < 0.001 by One-Way ANOVA with Bonferroni correction.


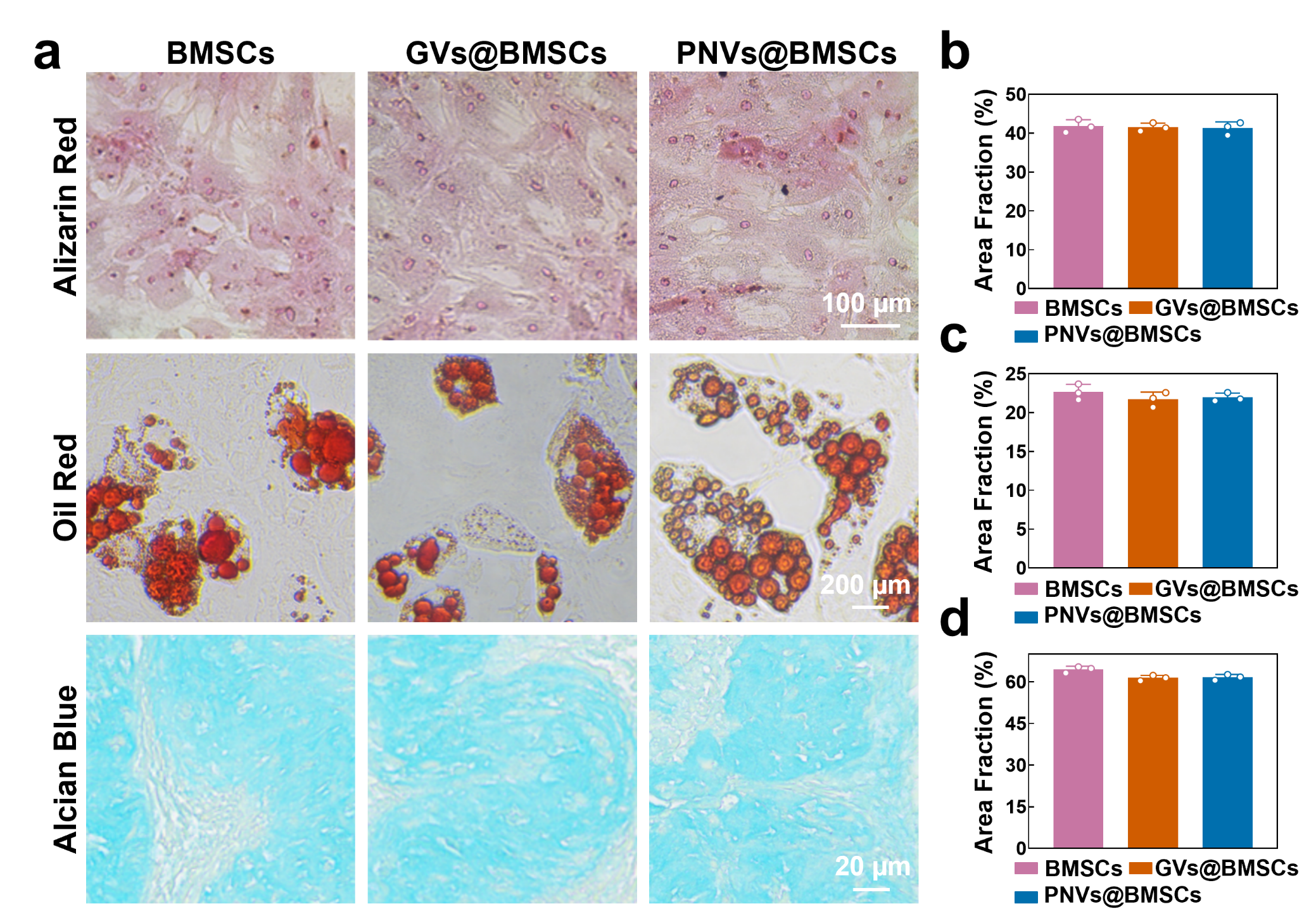


**Figure S7.** The impact of GVs and PNVs on the differentiation capacity of BMSCs. a) Alizarin red (pink), oil red O (red), and Alcian blue (blue) staining were used to quantify the osteogenic, adipogenic, and chondrogenic capacities of BMSCs, GVs@BMSCs, and PNVs@BMSCs. b-d) Quantitative analysis of osteocytes (b), adipocytes (c), and chondrocytes (d) in different groups (*n* = 3).


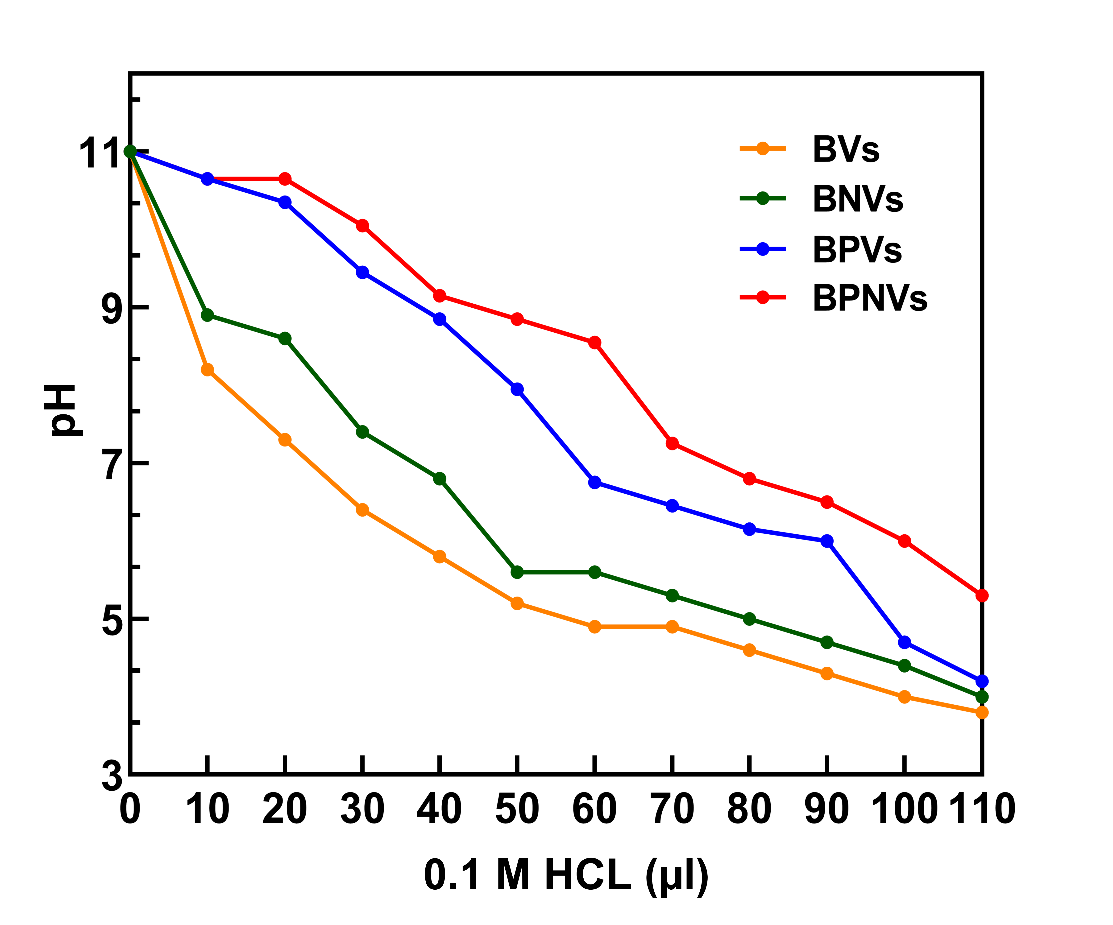


**Figure S8.** Comparative study of buffering capacity of BVs, BNVs, BPVs, and BPNVs by acid-base titration experiment.


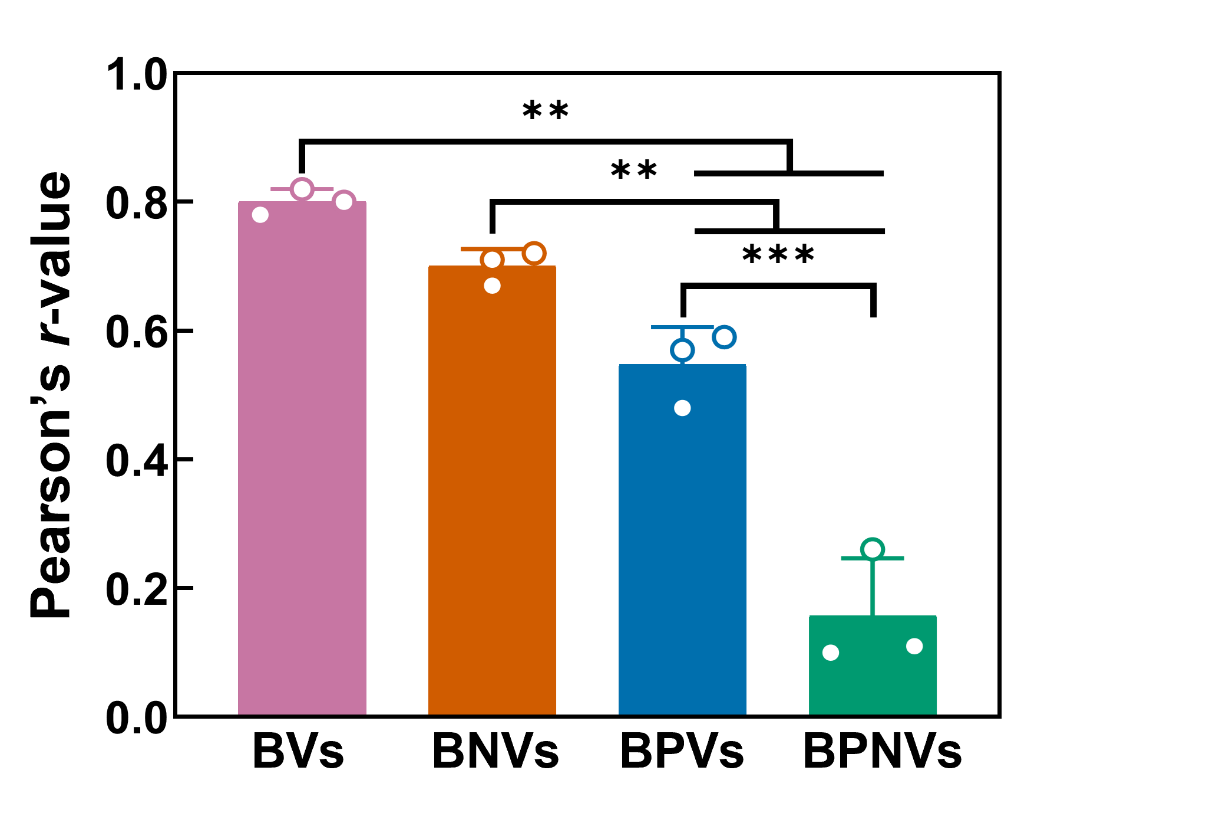


**Figure S9.** Pearson's *r*-value for lysosome colocalization with BVs, BNVs, BPVs, or BPNVs (*n* = 3). ** *p* <0.01, ****p* < 0.001 by One-Way ANOVA with Bonferroni correction.


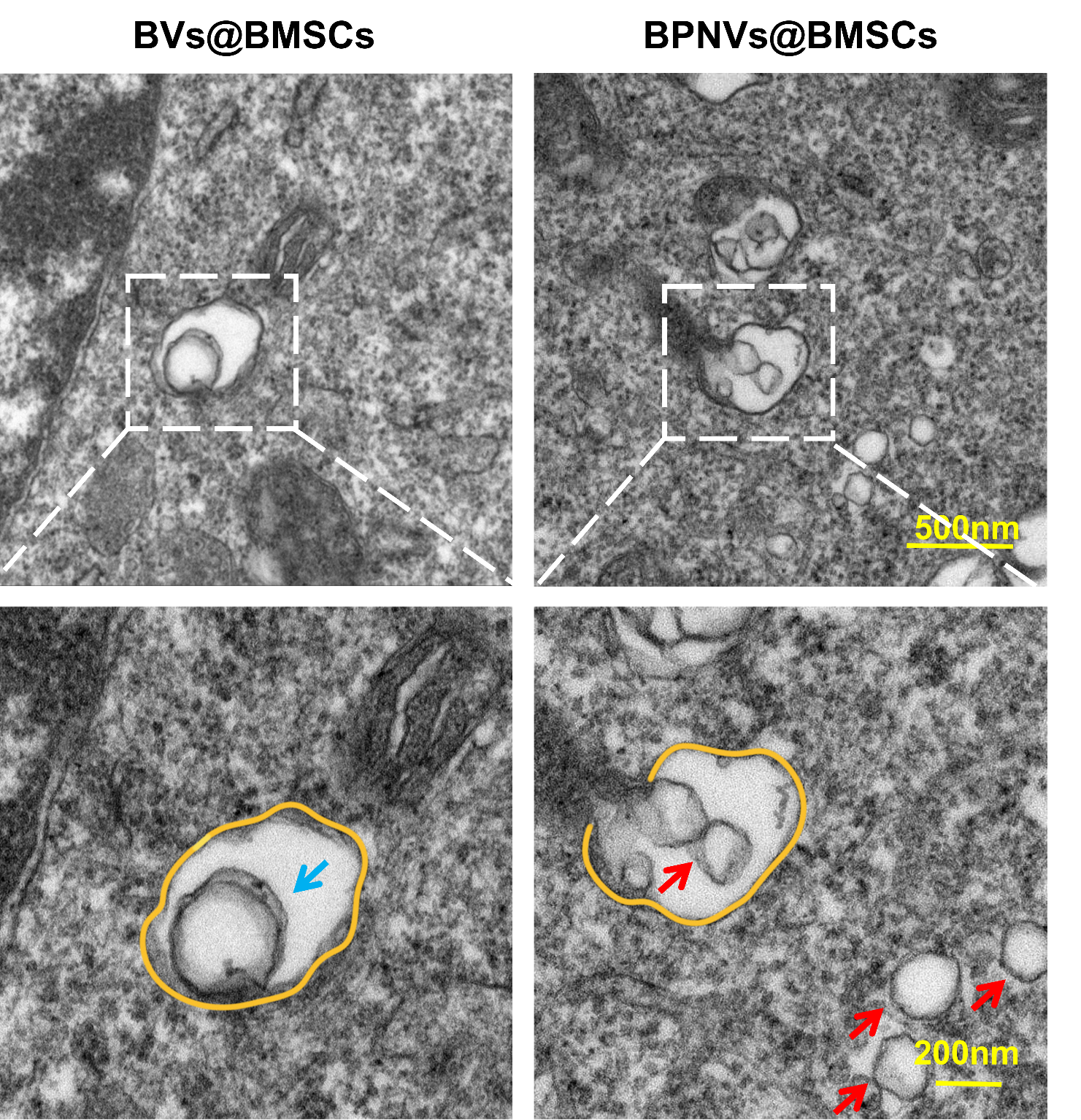


**Figure S10.** Morphological analysis of the lysosomal escape function. TEM images of BVs@BMSCs and BPNVs@BMSCs. Yellow line: lysosomal membrane, blue arrow: BVs, red arrow: BPNVs.


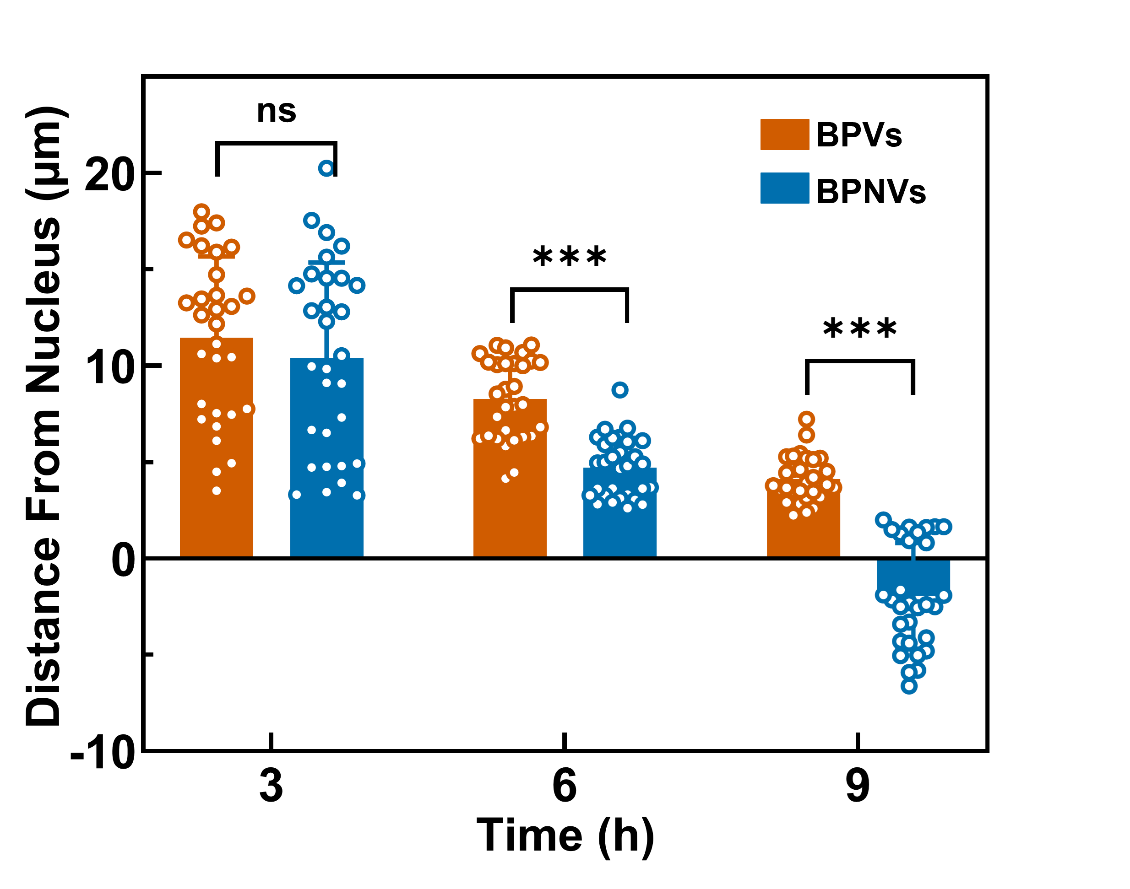


**Figure S11.** Quantitative analysis of the distance between BPVs or BPNVs and the nuclear membrane after co-incubation for 3,6,9 h (*n* = 30). ****p* < 0.001 by Paired *t*-test; ns = no significance.


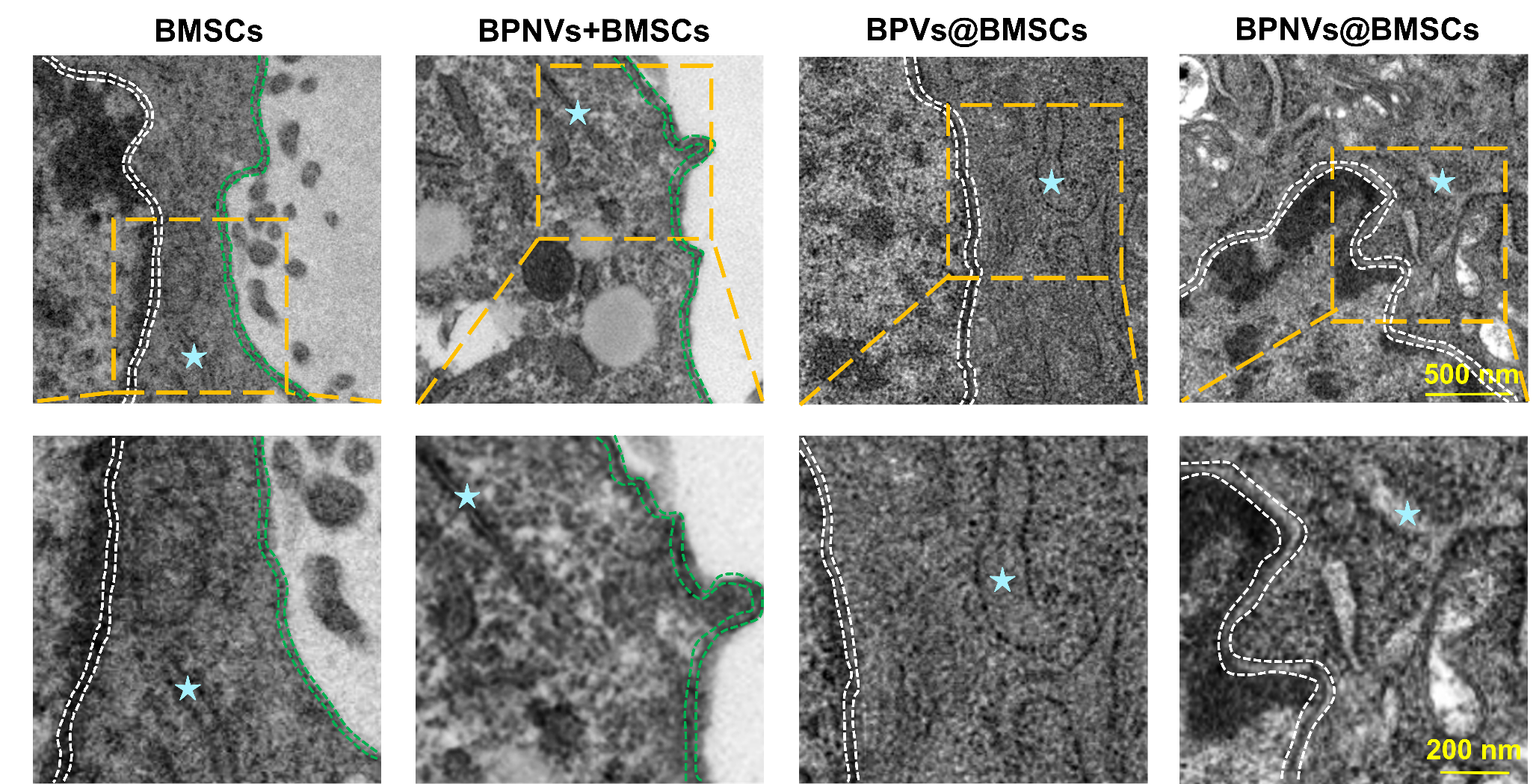


**Figure S12.** TEM images of BMSCs, BPNVs + BMSCs, BPVs@BMSCs, or BPNVs@BMSCs obtained either 8 hours after or without LIPUS sonication. White dotted line: nuclear membrane, green dotted line: cell membrane, blue stars: endoplasmic reticulum.


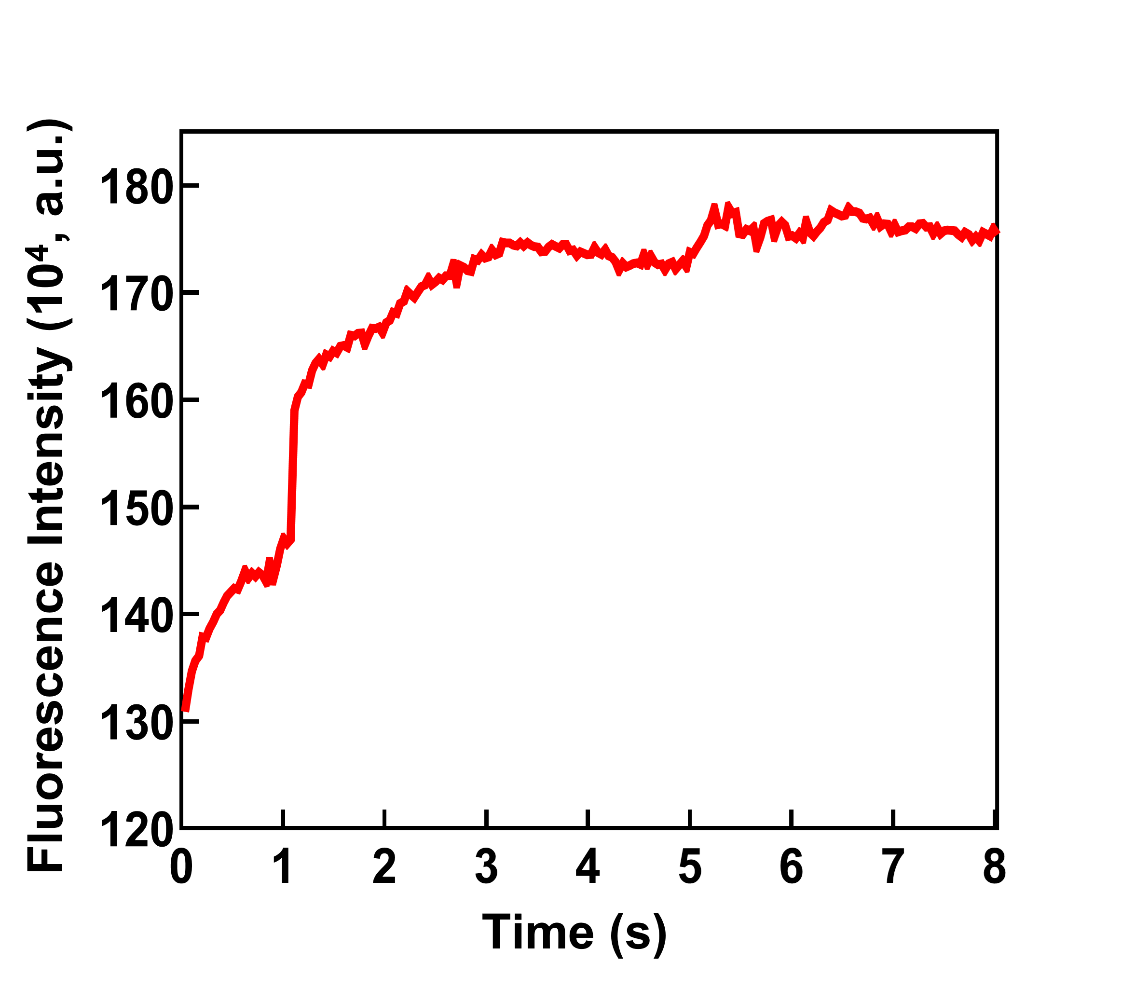


**Figure S13.** Quantitative analysis of plasmid fluorescence intensity within the nucleus over time of BPNVs@BMSCs after sonication.





**Figure S14.** Screening of ultrasonic parameters for gene transfection. a) Expression of EGFP in BPNVs@BMSCs after LIPUS sonication at different sound pressures (100, 110, 120, 130 mVpp), 20% duty cycle for 2.5 min. b) The gene transfection efficacy of BPNVs@BMSCs was assessed quantitatively by flow cytometry. **c,** Percentages of viable BPNVs@BMSCs after LIPUS sonication at different sound pressures (100, 110, 120, 130 mVpp), 20% duty cycle for 2.5 min. d) Expression of EGFP in BPNVs@BMSCs after LIPUS sonication at 120 mVpp, 20% duty cycle for 2, 2.5, 3, 3.5 min. e) The gene transfection efficacy of BPNVs@BMSCs was assessed quantitatively by flow cytometry. f) Percentages of viable BPNVs@BMSCs after LIPUS sonication at 120 mVpp, 20% duty cycle for 2, 2.5, 3, 3.5 min (*n* = 3). **p* <0.5, ** *p* <0.01, ****p* < 0.001 by One-Way ANOVA with Bonferroni correction.


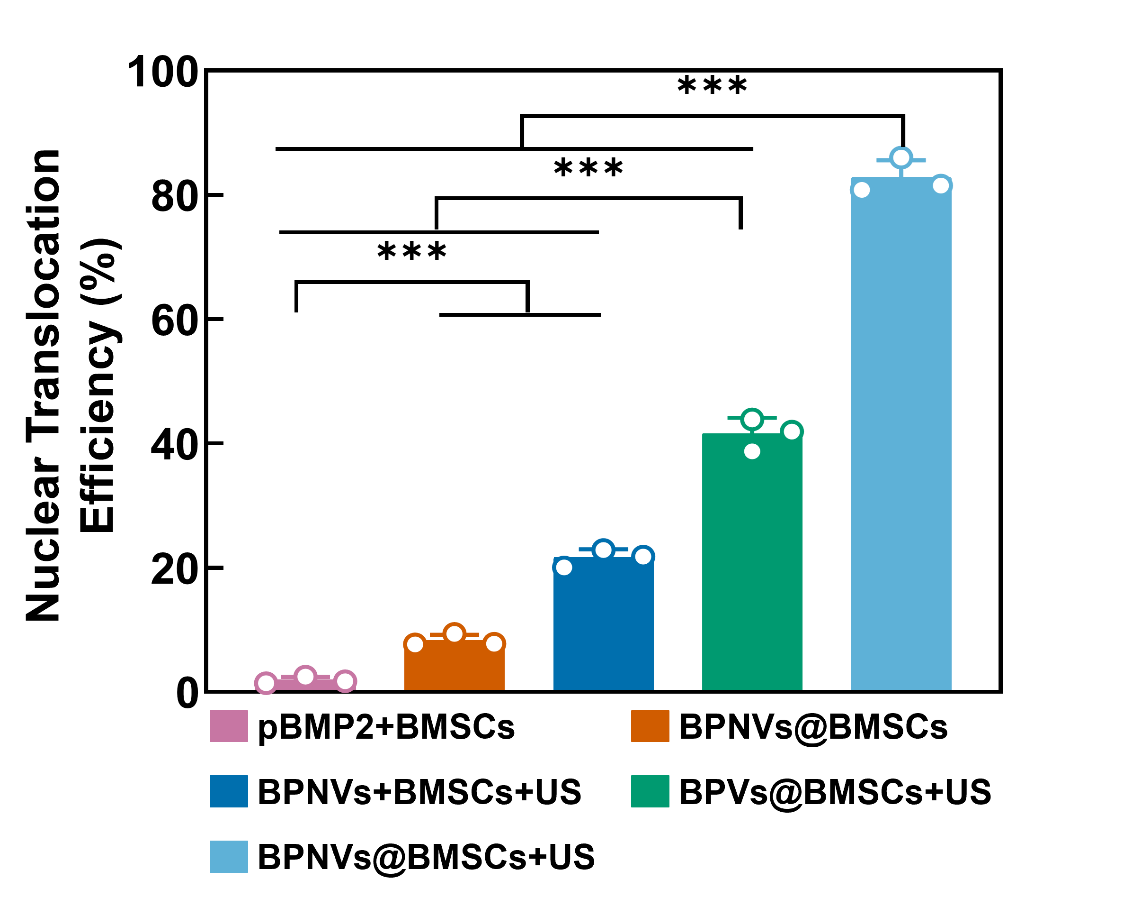


**Figure S15.** Quantitative analysis of plasmid nuclear translocation efficiency (*n* = 3). ****p* < 0.001 by One-Way ANOVA with Bonferroni correction.


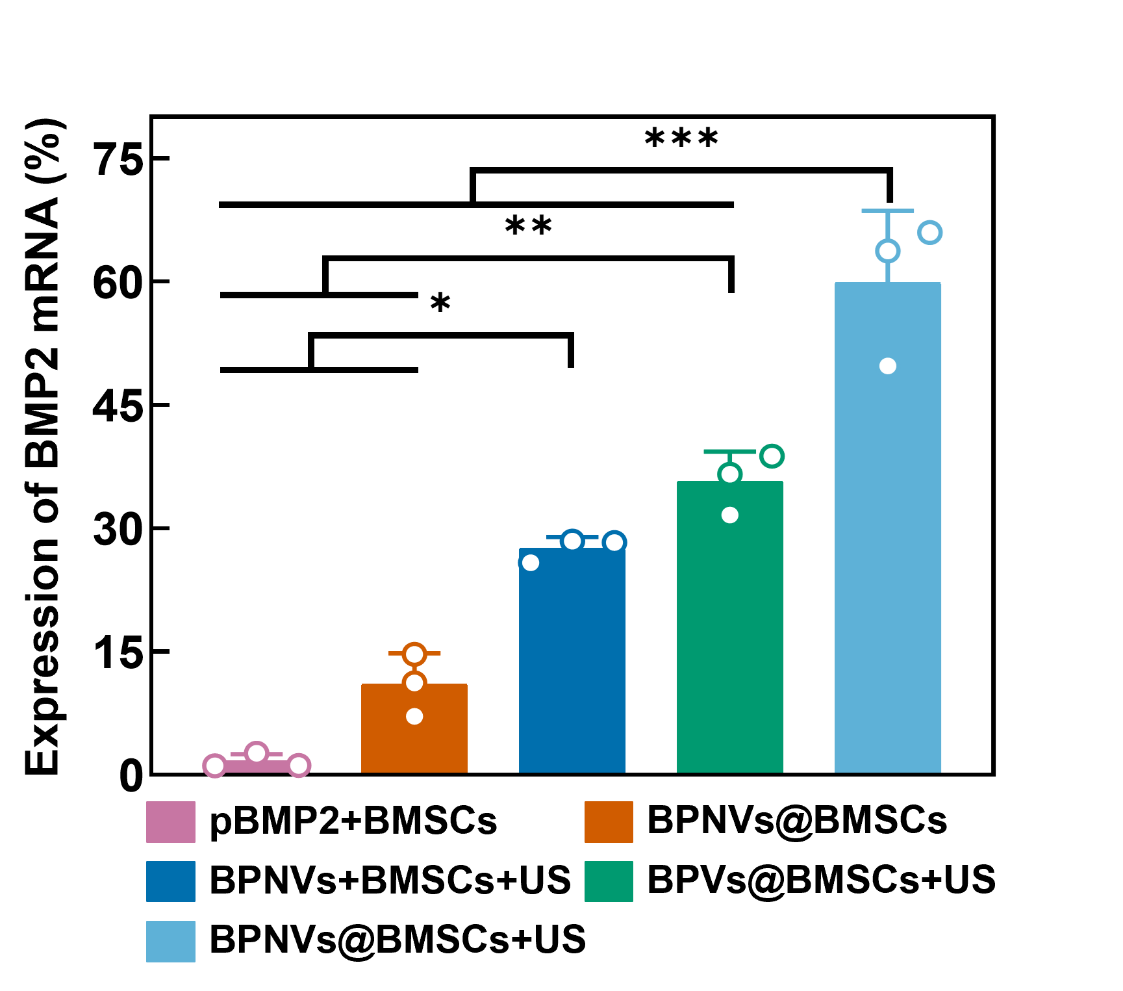


**Figure S16.** Detection of foreign gene expression by qRT-PCR. The relative mRNA expression of BMP2 in each group was determined 48 hours after gene transfection (*n* = 3). **p* <0.5, ** *p* <0.01, ****p* < 0.001 by One-Way ANOVA with Bonferroni correction.


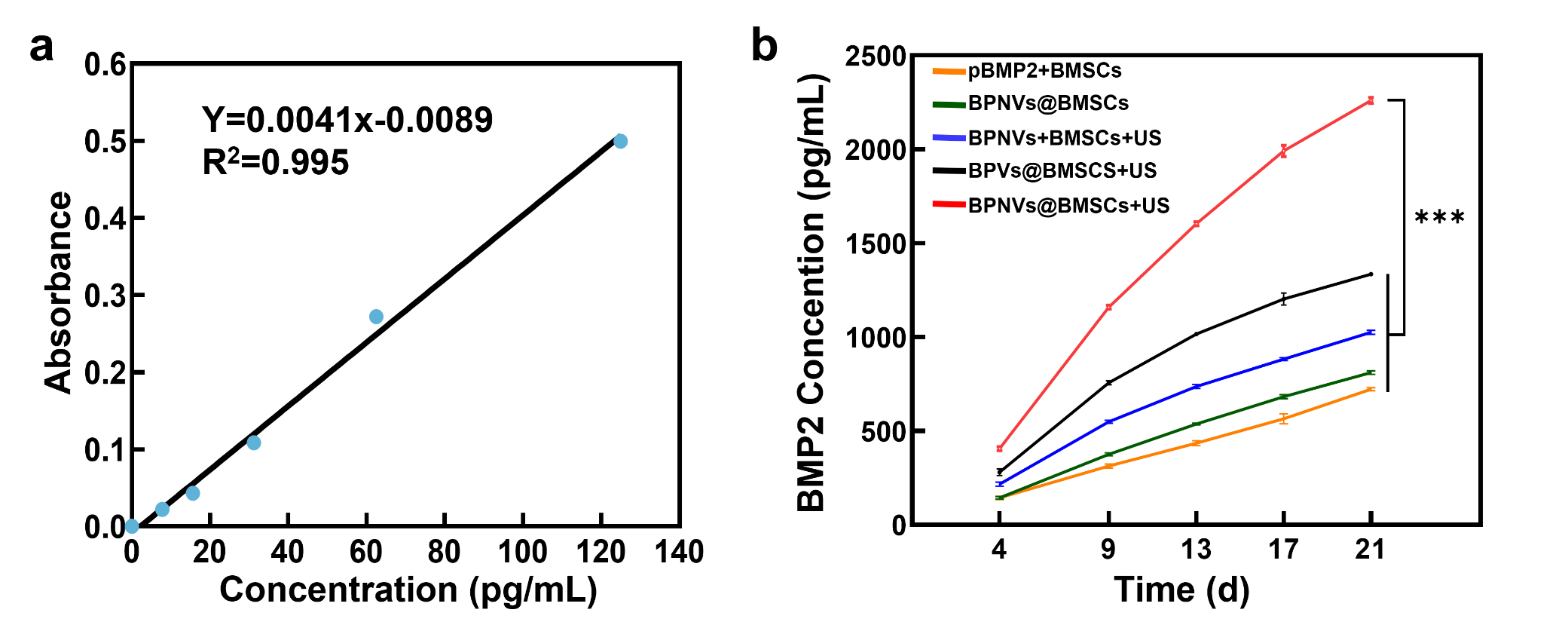


**Figure S17.** Detection of foreign gene protein expression by ELISA. a) Standard curve for the BMP2. b) Analysis of BMP2 concentration in the supernatant of each group (*n* = 3). ****p* < 0.001 by One-Way ANOVA with Bonferroni correction.


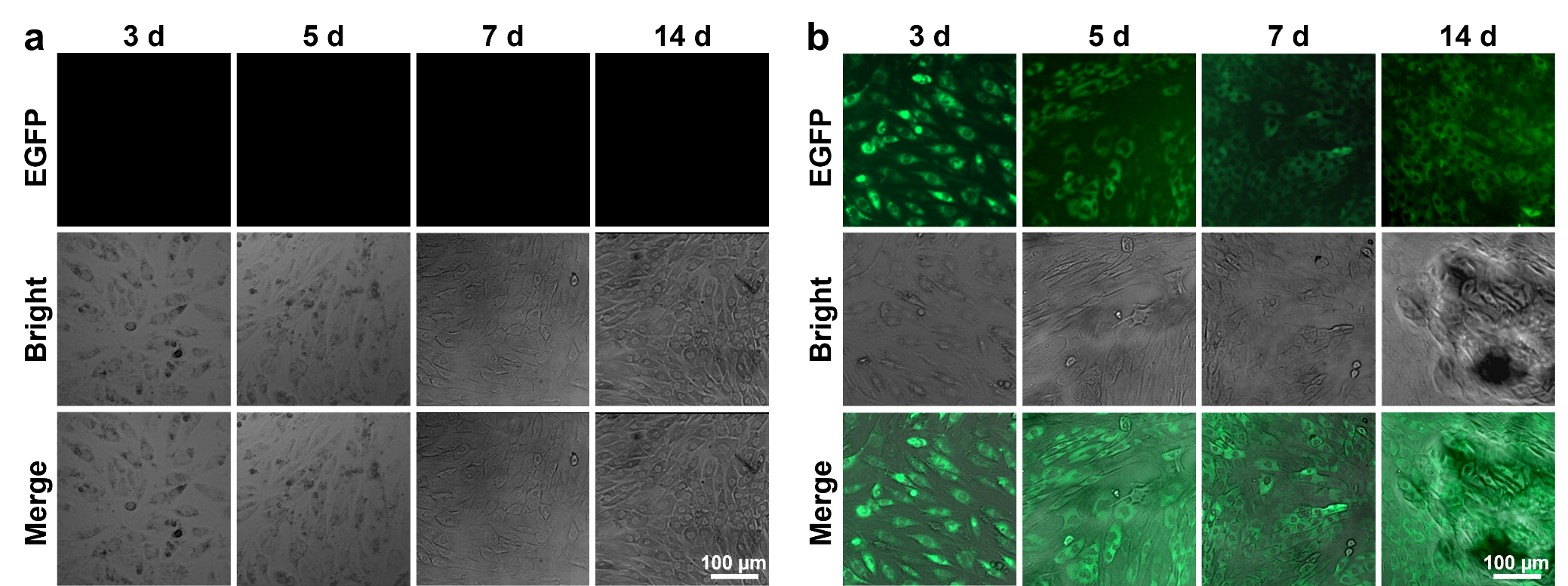


**Figure S18.** Fluorescence microscope images of morphological changes of osteogenesis in BMSCs. a,b) Expression of the EGFP reporter gene and mineralized nodule formation in the pBMP2+BMSCs group (a) and the BPNVs@BMSCs+US group (b) from 3 to 14 days after gene transfection.


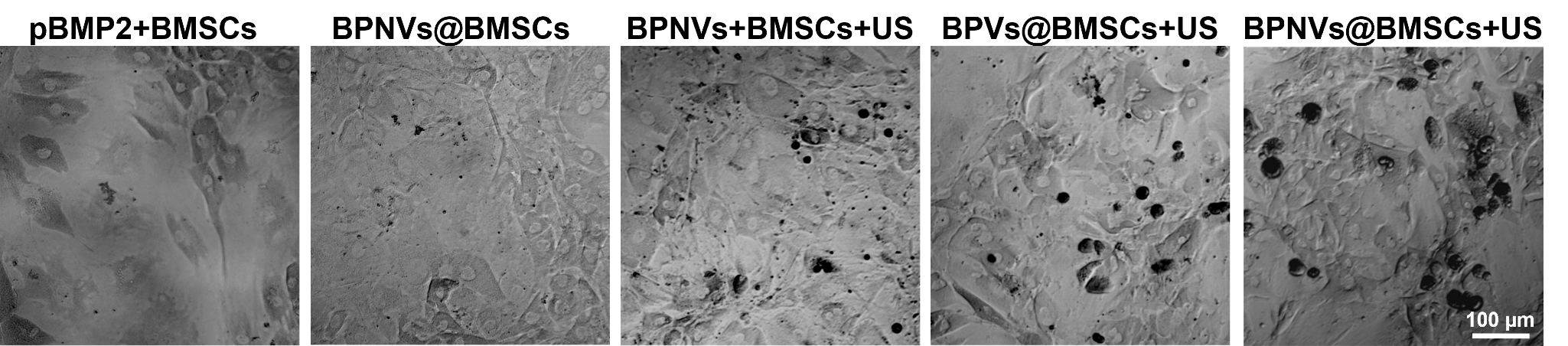


**Figure S19.** Evaluation of the osteogenic ability of BMSCs. Mineralized nodules were observed using a light microscope after 21 d of incubation in osteogenic differentiation media for each group.


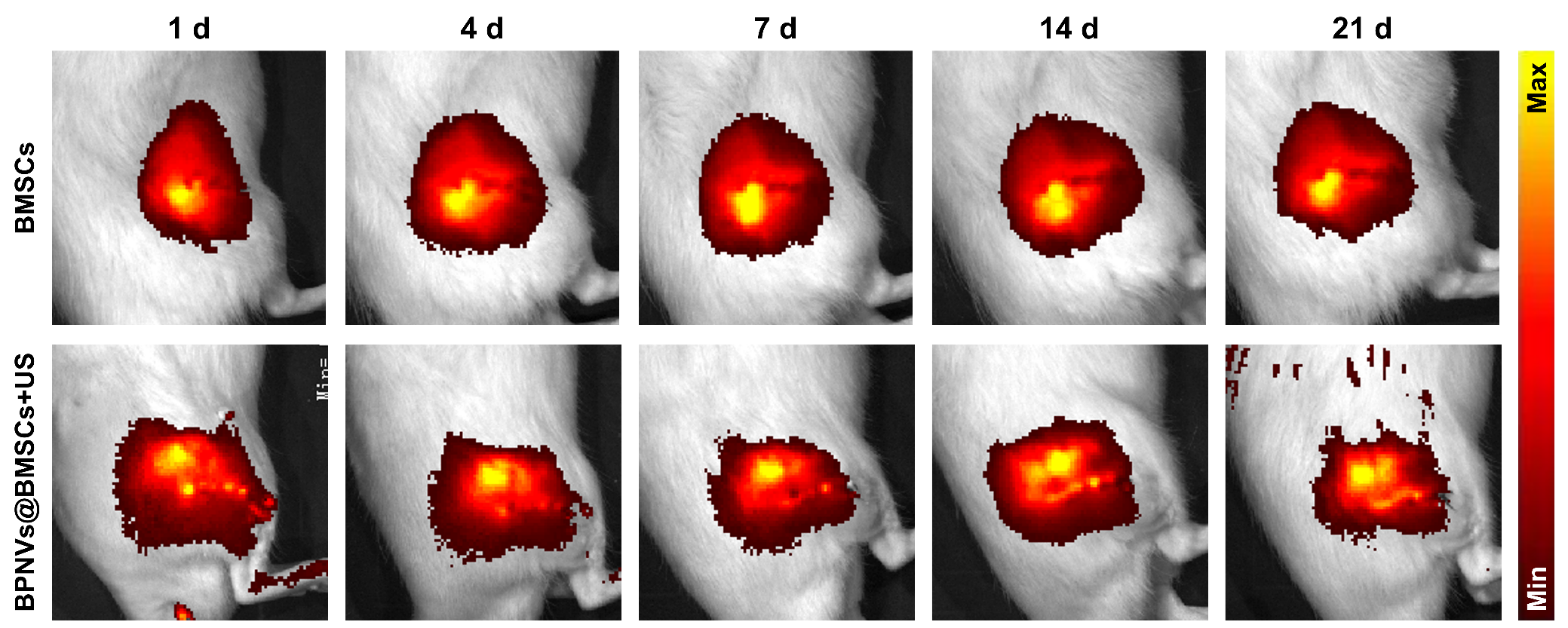


**Figure S20.** *In vivo* fluorescence images of the rats with bone defects at various times after injection of BMSsCs and BPNVs@BMSCs.


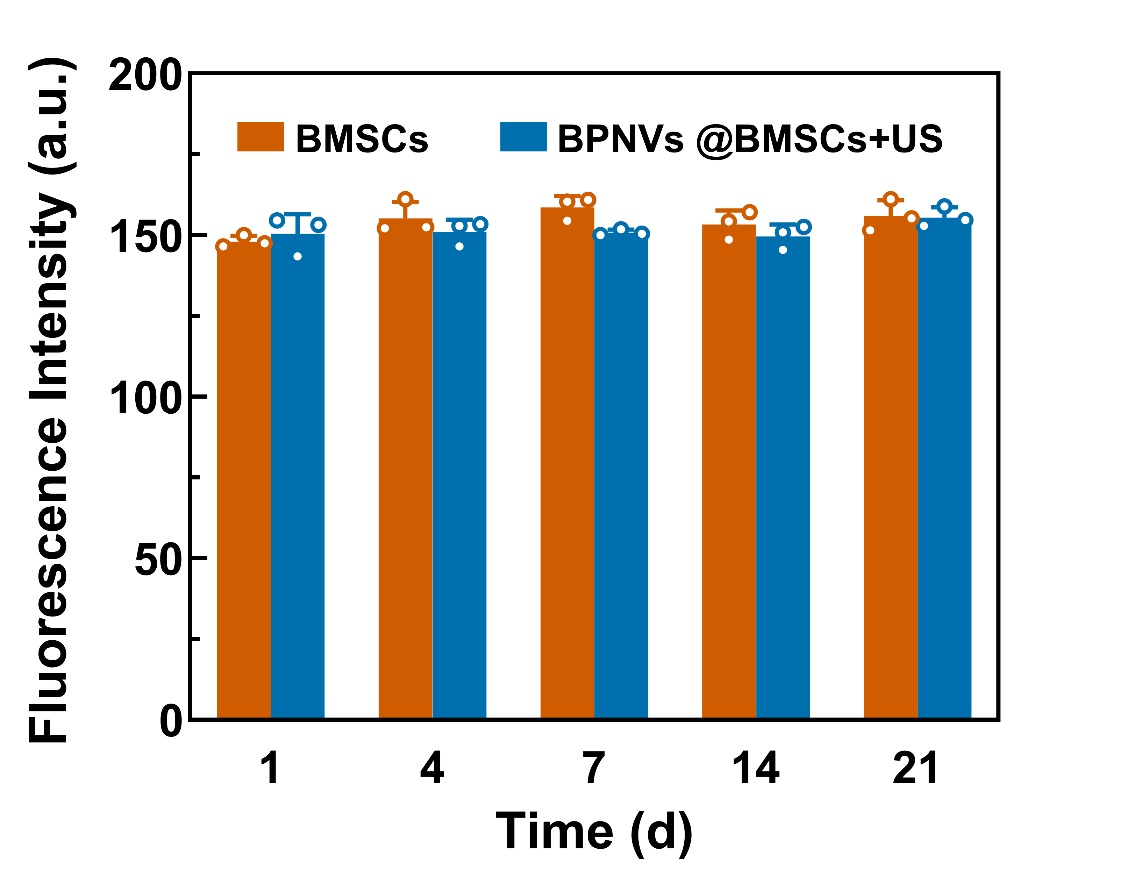


**Figure S21.** Fluorescence intensity of the rats with bone defects at various times after injection of BMSCs and BPNVs@BMSCs. Analysis of variance of factorial design.


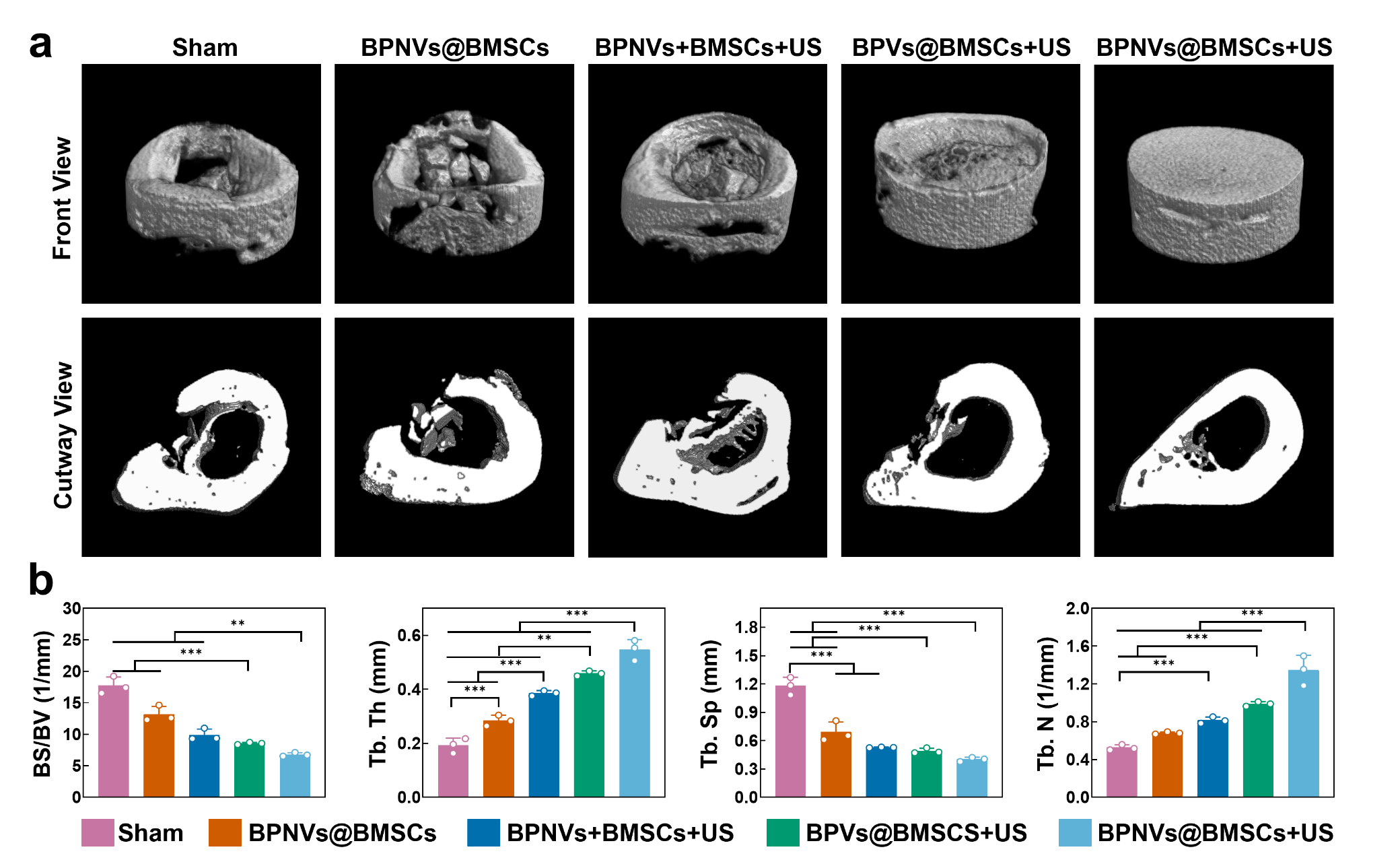


**Figure S22.** Micro-CT analysis of engineered BMSCs on repairment of femur defects. a) The three-dimensional reconstruction images of femur abnormalities in rats from various treatment groups. b) The bone-related parameters based on the micro-CT analysis, embracing BS/BV, Tb. Th, Tb. Sp and Tb. N (*n* = 3). ** *p* <0.01, ****p* < 0.001 by One-Way ANOVA with Bonferroni correction.


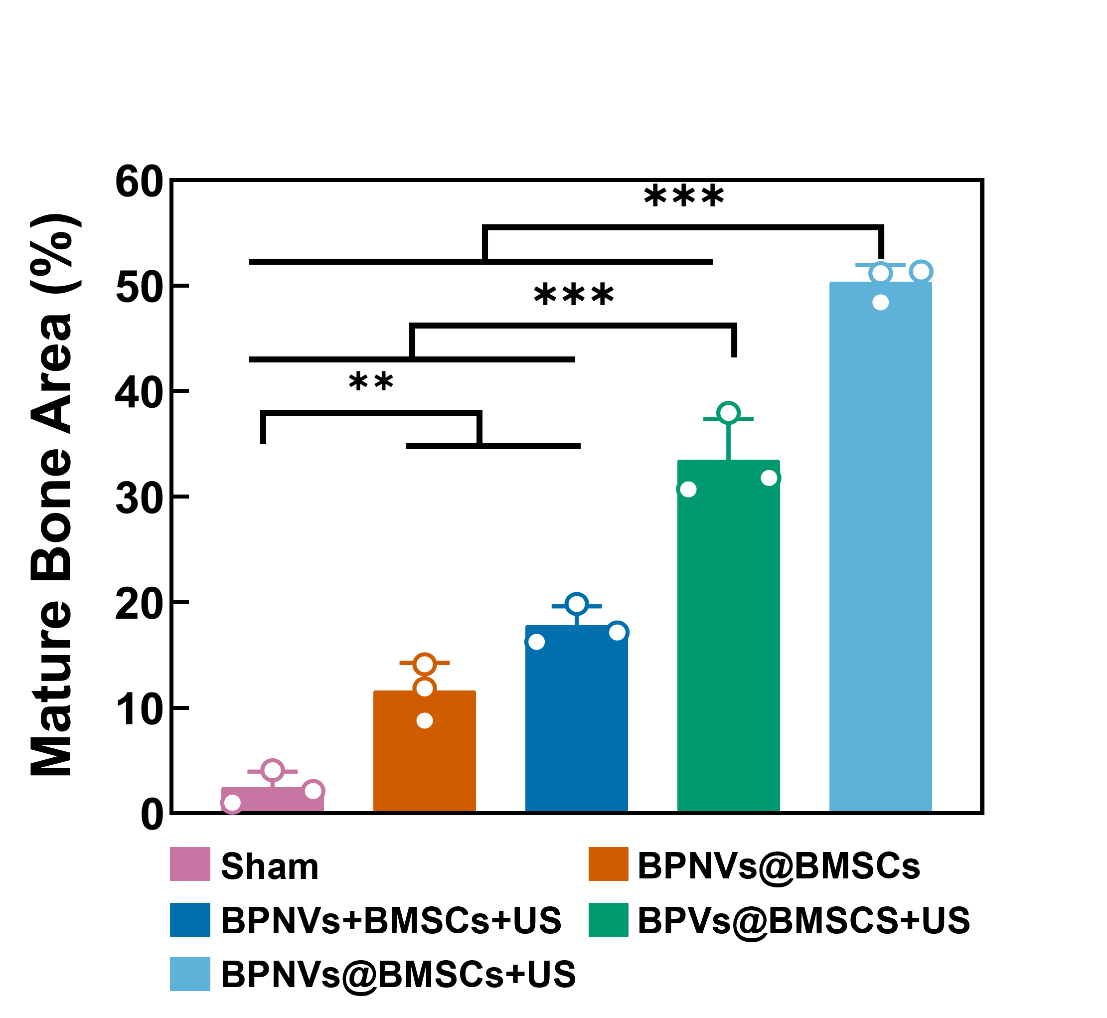


**Figure S23.** Quantitative analysis of mature bone in Masson staining (*n* = 3). ** *p* <0.01, ****p* < 0.001 by One-Way ANOVA with Bonferroni correction.


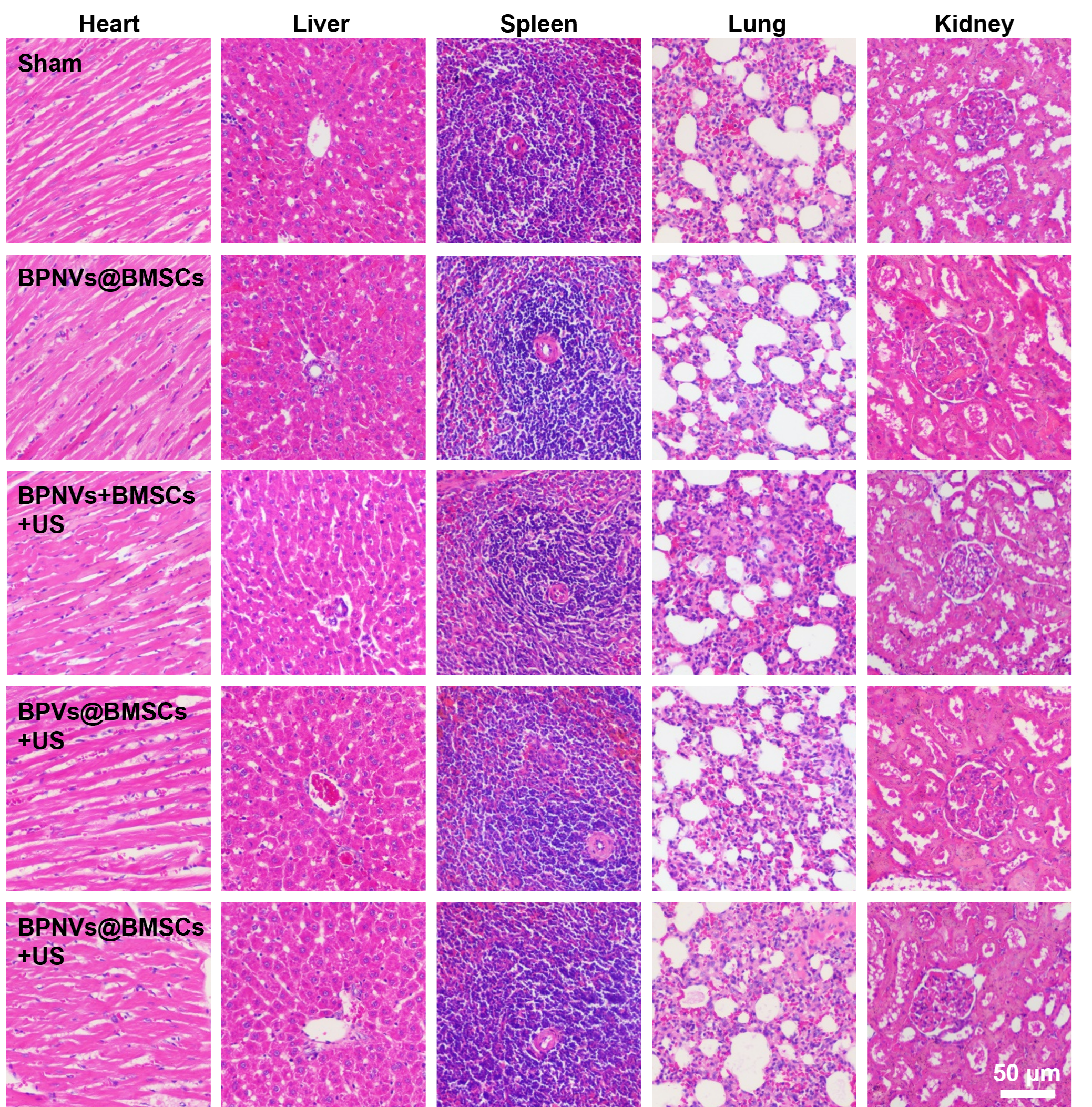


**Figure S24.** Representative H&E-stained sections of organs, including heart, liver, spleen, lung, and kidney, excised from rats after different treatments.
